# Supplementary material for: Corythauma ayyari (Insecta, Heteroptera, Tingidae) depends on its host plant to spread in Europe
Source: PLoS One. 2024 Mar 26;19(3):e0295102. doi: 10.1371/journal.pone.0295102 (PMC10965059; doi:10.1371/journal.pone.0295102)
Supplement: S1 Data — GPS coordinates of Jasminum officinale (Joff), J. grandiflorum (Jgra), J. multiflorum (Jmul), J. sambac (Jsam) and C. ayyari (Cay) occurrences after cleaning (see method section), used for analyses. (DOCX) [file pone.0295102.s001.docx]

S1 Data.

| Species | | Longitude | | Latitude | |
| --- | --- | --- | --- | --- | --- |
| Joff | | -9.417213 | | 38.90805 | |
| Joff | | -9.191108 | | 38.89534 | |
| Joff | | -9.179323 | | 38.586166 | |
| Joff | | -9.175705 | | 38.481128 | |
| Joff | | -9.171063 | | 39.417068 | |
| Joff | | -9.165417 | | 38.775614 | |
| Joff | | -9.155215 | | 38.729085 | |
| Joff | | -9.137822 | | 38.757047 | |
| Joff | | -8.853878 | | 52.339332 | |
| Joff | | -8.80366 | | 37.317558 | |
| Joff | | -8.610167 | | 41.163017 | |
| Joff | | -8.606629 | | 41.159047 | |
| Joff | | -8.605325 | | 41.161631 | |
| Joff | | -8.568098 | | 37.125366 | |
| Joff | | -8.37723 | | 40.855265 | |
| Joff | | -8.283989 | | 39.926701 | |
| Joff | | -8.234205 | | 39.756914 | |
| Joff | | -7.958797 | | 37.54293 | |
| Joff | | -7.928708 | | 37.018209 | |
| Joff | | -7.651389 | | 37.125278 | |
| Joff | | -7.577392 | | 37.928425 | |
| Joff | | -7.411488 | | 41.263 | |
| Joff | | -7 | | 38.8 | |
| Joff | | -6.97 | | 38.88 | |
| Joff | | -6.727435 | | 37.921225 | |
| Joff | | -6.58 | | 38.52 | |
| Joff | | -6.306294 | | 49.919157 | |
| Joff | | -6.276414 | | 57.01344 | |
| Joff | | -5.98875 | | 37.374592 | |
| Joff | | -5.274639 | | 36.575146 | |
| Joff | | -4.782687 | | 37.875488 | |
| Joff | | -4.673952 | | 48.357301 | |
| Joff | | -4.571686 | | 54.176135 | |
| Joff | | -4.555313 | | 48.292469 | |
| Joff | | -4.5333 | | 41.1 | |
| Joff | | -4.41908 | | 36.7199 | |
| Joff | | -4.416 | | 36.718 | |
| Joff | | -4.267299 | | 48.291223 | |
| Joff | | -4.209541 | | 48.187824 | |
| Joff | | -4.208038 | | 48.489666 | |
| Joff | | -4.145296 | | 47.92906 | |
| Joff | | -3.95 | | 36.91 | |
| Joff | | -3.8425 | | 48.5775 | |
| Joff | | -3.823941 | | 48.586826 | |
| Joff | | -3.617007 | | 55.903478 | |
| Joff | | -3.61202 | | 40.87122 | |
| Joff | | -3.591285 | | 37.1768 | |
| Joff | | -3.562868 | | 48.714569 | |
| Joff | | -3.415 | | 48.279722 | |
| Joff | | -3.382851 | | 51.686108 | |
| Joff | | -3.374396 | | 51.645744 | |
| Joff | | -3.35671 | | 48.652217 | |
| Joff | | -3.29362 | | 48.624991 | |
| Joff | | -3.250103 | | 48.240362 | |
| Joff | | -3.225066 | | 51.467561 | |
| Joff | | -3.223121 | | 48.155344 | |
| Joff | | -3.22 | | 42.85 | |
| Joff | | -3.204644 | | 47.664489 | |
| Joff | | -3.188709 | | 55.979505 | |
| Joff | | -3.155021 | | 47.334402 | |
| Joff | | -3.14 | | 37.3 | |
| Joff | | -3.120266 | | 48.734312 | |
| Joff | | -3.014268 | | 47.714144 | |
| Joff | | -2.85178 | | 56.018971 | |
| Joff | | -2.843934 | | 54.167836 | |
| Joff | | -2.822111 | | 47.662003 | |
| Joff | | -2.821247 | | 51.48823 | |
| Joff | | -2.813104 | | 48.570168 | |
| Joff | | -2.756667 | | 48.519722 | |
| Joff | | -2.744952 | | 53.77302 | |
| Joff | | -2.74 | | 42.97 | |
| Joff | | -2.719523 | | 51.442582 | |
| Joff | | -2.687239 | | 53.737809 | |
| Joff | | -2.677622 | | 53.359949 | |
| Joff | | -2.667778 | | 53.706696 | |
| Joff | | -2.644557 | | 47.573672 | |
| Joff | | -2.644556 | | 47.573673 | |
| Joff | | -2.62863 | | 53.695623 | |
| Joff | | -2.627373 | | 51.449804 | |
| Joff | | -2.622514 | | 53.701813 | |
| Joff | | -2.621984 | | 53.665863 | |
| Joff | | -2.612984 | | 51.44988 | |
| Joff | | -2.602998 | | 51.447182 | |
| Joff | | -2.58409 | | 51.441036 | |
| Joff | | -2.561689 | | 53.684137 | |
| Joff | | -2.56 | | 42.54 | |
| Joff | | -2.541035 | | 51.450234 | |
| Joff | | -2.538761 | | 49.479562 | |
| Joff | | -2.526265 | | 53.270825 | |
| Joff | | -2.520648 | | 51.612308 | |
| Joff | | -2.50925 | | 47.527161 | |
| Joff | | -2.441606 | | 51.373151 | |
| Joff | | -2.420044 | | 48.440666 | |
| Joff | | -2.416 | | 43.113 | |
| Joff | | -2.377195 | | 51.37073 | |
| Joff | | -2.374754 | | 53.091619 | |
| Joff | | -2.362877 | | 46.719577 | |
| Joff | | -2.308255 | | 52.138892 | |
| Joff | | -2.24946 | | 48.619396 | |
| Joff | | -2.225435 | | 53.091996 | |
| Joff | | -2.223483 | | 53.495493 | |
| Joff | | -2.214759 | | 47.709945 | |
| Joff | | -2.184812 | | 48.42601 | |
| Joff | | -2.157884 | | 46.924096 | |
| Joff | | -2.131018 | | 47.114521 | |
| Joff | | -2.127492 | | 47.120322 | |
| Joff | | -2.111091 | | 47.115006 | |
| Joff | | -2.1075 | | 47.123333 | |
| Joff | | -2.1 | | 36.91 | |
| Joff | | -2.079444 | | 47.333333 | |
| Joff | | -2.074293 | | 52.031386 | |
| Joff | | -2.071432 | | 47.099721 | |
| Joff | | -2.070354 | | 47.113437 | |
| Joff | | -2.070242 | | 47.114311 | |
| Joff | | -2.046653 | | 48.451548 | |
| Joff | | -2.028093 | | 48.380112 | |
| Joff | | -1.980291 | | 48.643488 | |
| Joff | | -1.957615 | | 52.103326 | |
| Joff | | -1.888689 | | 46.850222 | |
| Joff | | -1.884722 | | 46.882222 | |
| Joff | | -1.840825 | | 46.833821 | |
| Joff | | -1.836186 | | 46.600616 | |
| Joff | | -1.793775 | | 47.327806 | |
| Joff | | -1.793769 | | 47.327818 | |
| Joff | | -1.79 | | 41.57 | |
| Joff | | -1.781778 | | 47.270501 | |
| Joff | | -1.762299 | | 43.365312 | |
| Joff | | -1.754267 | | 43.370142 | |
| Joff | | -1.75 | | 37.4 | |
| Joff | | -1.747624 | | 43.373903 | |
| Joff | | -1.74228 | | 48.49013 | |
| Joff | | -1.7 | | 43.39 | |
| Joff | | -1.679382 | | 48.144518 | |
| Joff | | -1.671102 | | 47.202248 | |
| Joff | | -1.658908 | | 48.106615 | |
| Joff | | -1.652886 | | 43.396868 | |
| Joff | | -1.629207 | | 47.27461 | |
| Joff | | -1.624415 | | 49.632805 | |
| Joff | | -1.614532 | | 49.632379 | |
| Joff | | -1.61 | | 43.15 | |
| Joff | | -1.5825 | | 47.194167 | |
| Joff | | -1.582104 | | 51.658577 | |
| Joff | | -1.580409 | | 51.626076 | |
| Joff | | -1.58 | | 43.48 | |
| Joff | | -1.579287 | | 47.120715 | |
| Joff | | -1.577951 | | 51.624773 | |
| Joff | | -1.57572 | | 47.50117 | |
| Joff | | -1.554698 | | 47.171502 | |
| Joff | | -1.543439 | | 47.18985 | |
| Joff | | -1.53506 | | 47.51814 | |
| Joff | | -1.53402 | | 47.221799 | |
| Joff | | -1.526013 | | 51.411368 | |
| Joff | | -1.516389 | | 47.241667 | |
| Joff | | -1.516208 | | 47.200309 | |
| Joff | | -1.515438 | | 47.239041 | |
| Joff | | -1.509904 | | 51.414494 | |
| Joff | | -1.475556 | | 43.491111 | |
| Joff | | -1.466722 | | 43.494359 | |
| Joff | | -1.46 | | 43.39 | |
| Joff | | -1.459286 | | 43.500951 | |
| Joff | | -1.45 | | 43.48 | |
| Joff | | -1.449167 | | 43.469167 | |
| Joff | | -1.448024 | | 43.43673 | |
| Joff | | -1.431832 | | 51.606282 | |
| Joff | | -1.421686 | | 43.647435 | |
| Joff | | -1.388827 | | 43.355145 | |
| Joff | | -1.388231 | | 43.542846 | |
| Joff | | -1.357452 | | 53.72747 | |
| Joff | | -1.355121 | | 54.511936 | |
| Joff | | -1.349288 | | 43.646373 | |
| Joff | | -1.348616 | | 51.759422 | |
| Joff | | -1.343275 | | 51.665065 | |
| Joff | | -1.340213 | | 51.393512 | |
| Joff | | -1.323205 | | 34.91419 | |
| Joff | | -1.316548 | | 51.395885 | |
| Joff | | -1.313739 | | 46.720208 | |
| Joff | | -1.290113 | | 51.660305 | |
| Joff | | -1.28782 | | 54.562784 | |
| Joff | | -1.287426 | | 54.563187 | |
| Joff | | -1.280354 | | 51.774318 | |
| Joff | | -1.27645 | | 47.093224 | |
| Joff | | -1.273685 | | 51.75188 | |
| Joff | | -1.263402 | | 51.399915 | |
| Joff | | -1.260966 | | 51.399432 | |
| Joff | | -1.260627 | | 51.746943 | |
| Joff | | -1.260301 | | 51.759808 | |
| Joff | | -1.257324 | | 51.564721 | |
| Joff | | -1.256825 | | 51.743835 | |
| Joff | | -1.2538 | | 51.735778 | |
| Joff | | -1.238092 | | 51.400481 | |
| Joff | | -1.237186 | | 51.571452 | |
| Joff | | -1.235272 | | 44.198042 | |
| Joff | | -1.233135 | | 51.567811 | |
| Joff | | -1.220231 | | 51.730777 | |
| Joff | | -1.202221 | | 48.344211 | |
| Joff | | -1.195363 | | 43.849764 | |
| Joff | | -1.178316 | | 51.395114 | |
| Joff | | -1.171303 | | 54.512614 | |
| Joff | | -1.163944 | | 51.642274 | |
| Joff | | -1.154896 | | 51.521677 | |
| Joff | | -1.149 | | 37.992 | |
| Joff | | -1.14065 | | 44.765007 | |
| Joff | | -1.118327 | | 37.979795 | |
| Joff | | -1.088046 | | 46.154561 | |
| Joff | | -1.05863 | | 51.401291 | |
| Joff | | -1.055907 | | 51.40786 | |
| Joff | | -1.048084 | | 51.4488 | |
| Joff | | -1.045321 | | 51.44946 | |
| Joff | | -1.043759 | | 50.788844 | |
| Joff | | -1.04332 | | 51.373697 | |
| Joff | | -1.038239 | | 45.981703 | |
| Joff | | -1.030975 | | 51.471139 | |
| Joff | | -1.03 | | 39.96 | |
| Joff | | -0.982348 | | 51.457869 | |
| Joff | | -0.976154 | | 45.938573 | |
| Joff | | -0.969851 | | 51.447779 | |
| Joff | | -0.941281 | | 50.784663 | |
| Joff | | -0.91548 | | 51.424899 | |
| Joff | | -0.912745 | | 51.472845 | |
| Joff | | -0.9125 | | 48.874722 | |
| Joff | | -0.887537 | | 46.215442 | |
| Joff | | -0.873519 | | 51.560118 | |
| Joff | | -0.83075 | | 51.519471 | |
| Joff | | -0.817462 | | 51.37124 | |
| Joff | | -0.817232 | | 51.405254 | |
| Joff | | -0.814537 | | 51.393268 | |
| Joff | | -0.811339 | | 51.376339 | |
| Joff | | -0.790339 | | 51.432244 | |
| Joff | | -0.789298 | | 51.436261 | |
| Joff | | -0.785629 | | 51.365681 | |
| Joff | | -0.773344 | | 51.392718 | |
| Joff | | -0.763707 | | 48.071109 | |
| Joff | | -0.762184 | | 51.51375 | |
| Joff | | -0.755771 | | 46.127896 | |
| Joff | | -0.753518 | | 47.877281 | |
| Joff | | -0.746665 | | 46.433514 | |
| Joff | | -0.746643 | | 46.433479 | |
| Joff | | -0.740917 | | 51.424143 | |
| Joff | | -0.740512 | | 51.526189 | |
| Joff | | -0.739819 | | 44.900323 | |
| Joff | | -0.737707 | | 51.516061 | |
| Joff | | -0.736507 | | 51.525085 | |
| Joff | | -0.726881 | | 51.51928 | |
| Joff | | -0.71854 | | 51.509361 | |
| Joff | | -0.718055 | | 51.422376 | |
| Joff | | -0.714513 | | 51.441984 | |
| Joff | | -0.707359 | | 51.577467 | |
| Joff | | -0.703997 | | 51.515594 | |
| Joff | | -0.702947 | | 51.526679 | |
| Joff | | -0.700086 | | 43.642532 | |
| Joff | | -0.693056 | | 46.296944 | |
| Joff | | -0.67153 | | 51.40292 | |
| Joff | | -0.662914 | | 46.201303 | |
| Joff | | -0.6592 | | 44.840652 | |
| Joff | | -0.657579 | | 45.823797 | |
| Joff | | -0.65 | | 39.6 | |
| Joff | | -0.648788 | | 44.830367 | |
| Joff | | -0.640826 | | 51.390159 | |
| Joff | | -0.640219 | | 51.39659 | |
| Joff | | -0.639693 | | 51.408975 | |
| Joff | | -0.634865 | | 51.479354 | |
| Joff | | -0.633443 | | 44.832205 | |
| Joff | | -0.622145 | | 51.478037 | |
| Joff | | -0.62073 | | 47.267658 | |
| Joff | | -0.62 | | 39.32 | |
| Joff | | -0.616374 | | 51.470308 | |
| Joff | | -0.614099 | | 51.478877 | |
| Joff | | -0.613653 | | 44.841396 | |
| Joff | | -0.61354 | | 51.480705 | |
| Joff | | -0.608333 | | 47.478889 | |
| Joff | | -0.607858 | | 43.193655 | |
| Joff | | -0.6 | | 40.04 | |
| Joff | | -0.591995 | | 44.832841 | |
| Joff | | -0.589662 | | 44.841327 | |
| Joff | | -0.588751 | | 44.844463 | |
| Joff | | -0.586085 | | 51.461817 | |
| Joff | | -0.581273 | | 51.462398 | |
| Joff | | -0.580523 | | 51.451328 | |
| Joff | | -0.580328 | | 44.848263 | |
| Joff | | -0.5769 | | 44.865747 | |
| Joff | | -0.576111 | | 45.066111 | |
| Joff | | -0.575205 | | 44.803991 | |
| Joff | | -0.573743 | | 44.821007 | |
| Joff | | -0.571834 | | 44.828977 | |
| Joff | | -0.568828 | | 44.805736 | |
| Joff | | -0.568063 | | 47.499992 | |
| Joff | | -0.56599 | | 44.825326 | |
| Joff | | -0.565367 | | 44.815737 | |
| Joff | | -0.563069 | | 44.814262 | |
| Joff | | -0.554798 | | 44.768609 | |
| Joff | | -0.544774 | | 47.458405 | |
| Joff | | -0.530867 | | 47.447886 | |
| Joff | | -0.530808 | | 47.447993 | |
| Joff | | -0.525443 | | 47.477211 | |
| Joff | | -0.523056 | | 47.460278 | |
| Joff | | -0.518471 | | 47.434305 | |
| Joff | | -0.500261 | | 46.841896 | |
| Joff | | -0.5 | | 39.59 | |
| Joff | | -0.481632 | | 44.821993 | |
| Joff | | -0.471148 | | 47.468781 | |
| Joff | | -0.466313 | | 44.920087 | |
| Joff | | -0.46 | | 38.41 | |
| Joff | | -0.447639 | | 51.648038 | |
| Joff | | -0.446819 | | 46.311749 | |
| Joff | | -0.436952 | | 45.446963 | |
| Joff | | -0.432821 | | 43.337265 | |
| Joff | | -0.423641 | | 54.27548 | |
| Joff | | -0.421986 | | 51.679168 | |
| Joff | | -0.42084 | | 44.914028 | |
| Joff | | -0.418563 | | 43.254213 | |
| Joff | | -0.415134 | | 51.668285 | |
| Joff | | -0.409152 | | 44.773114 | |
| Joff | | -0.391109 | | 49.196243 | |
| Joff | | -0.39 | | 39.44 | |
| Joff | | -0.39 | | 39.48 | |
| Joff | | -0.38 | | 39.5 | |
| Joff | | -0.359349 | | 51.731362 | |
| Joff | | -0.357265 | | 49.285638 | |
| Joff | | -0.352588 | | 49.194675 | |
| Joff | | -0.348648 | | 45.695777 | |
| Joff | | -0.347587 | | 43.315063 | |
| Joff | | -0.344613 | | 51.738349 | |
| Joff | | -0.3413 | | 39.456653 | |
| Joff | | -0.332796 | | 49.242968 | |
| Joff | | -0.328998 | | 51.690471 | |
| Joff | | -0.29677 | | 51.718818 | |
| Joff | | -0.264856 | | 51.665262 | |
| Joff | | -0.25 | | 39.94 | |
| Joff | | -0.246612 | | 51.688371 | |
| Joff | | -0.224691 | | 43.53854 | |
| Joff | | -0.21963 | | 43.136805 | |
| Joff | | -0.215088 | | 51.643834 | |
| Joff | | -0.182778 | | 45.476389 | |
| Joff | | -0.1748 | | 44.9895 | |
| Joff | | -0.171743 | | 51.787042 | |
| Joff | | -0.166574 | | 51.735706 | |
| Joff | | -0.150254 | | 44.783286 | |
| Joff | | -0.150149 | | 44.783008 | |
| Joff | | -0.141944 | | 46.995556 | |
| Joff | | -0.130503 | | 51.535274 | |
| Joff | | -0.113611 | | 46.359444 | |
| Joff | | -0.071039 | | 42.990998 | |
| Joff | | -0.000905 | | 45.012095 | |
| Joff | | 0.008 | | 52.1 | |
| Joff | | 0.14 | | 40.97 | |
| Joff | | 0.146506 | | 43.064212 | |
| Joff | | 0.15 | | 40.79 | |
| Joff | | 0.152938 | | 45.650437 | |
| Joff | | 0.157883 | | 52.247118 | |
| Joff | | 0.17216 | | 44.50536 | |
| Joff | | 0.183333 | | 47.926389 | |
| Joff | | 0.19134 | | 48.00816 | |
| Joff | | 0.2075 | | 48.025833 | |
| Joff | | 0.20934 | | 44.703388 | |
| Joff | | 0.209513 | | 47.874789 | |
| Joff | | 0.269301 | | 49.697861 | |
| Joff | | 0.27 | | 40.79 | |
| Joff | | 0.303372 | | 46.629721 | |
| Joff | | 0.309509 | | 44.39256 | |
| Joff | | 0.320532 | | 45.601456 | |
| Joff | | 0.33 | | 40.85 | |
| Joff | | 0.342493 | | 44.137528 | |
| Joff | | 0.349722 | | 46.573611 | |
| Joff | | 0.379112 | | 46.56344 | |
| Joff | | 0.382296 | | 42.899911 | |
| Joff | | 0.390439 | | 46.644846 | |
| Joff | | 0.435021 | | 46.254109 | |
| Joff | | 0.479942 | | 43.723911 | |
| Joff | | 0.505209 | | 44.389575 | |
| Joff | | 0.519492 | | 46.728943 | |
| Joff | | 0.52003 | | 44.255802 | |
| Joff | | 0.528141 | | 43.059673 | |
| Joff | | 0.550953 | | 50.893089 | |
| Joff | | 0.561148 | | 46.40647 | |
| Joff | | 0.575991 | | 43.651665 | |
| Joff | | 0.58512 | | 44.678694 | |
| Joff | | 0.593183 | | 44.145699 | |
| Joff | | 0.61372 | | 42.91789 | |
| Joff | | 0.633277 | | 43.759223 | |
| Joff | | 0.644242 | | 47.362511 | |
| Joff | | 0.664129 | | 44.276353 | |
| Joff | | 0.677318 | | 46.229639 | |
| Joff | | 0.677778 | | 47.403056 | |
| Joff | | 0.68 | | 42.42 | |
| Joff | | 0.708791 | | 47.393696 | |
| Joff | | 0.715451 | | 47.287375 | |
| Joff | | 0.716092 | | 49.658194 | |
| Joff | | 0.744016 | | 49.620582 | |
| Joff | | 0.756111 | | 48.382778 | |
| Joff | | 0.783611 | | 48.975278 | |
| Joff | | 0.796827 | | 47.418266 | |
| Joff | | 0.809095 | | 43.085425 | |
| Joff | | 0.811104 | | 49.33201 | |
| Joff | | 0.83 | | 41.61 | |
| Joff | | 0.844722 | | 45.240556 | |
| Joff | | 0.874197 | | 51.973758 | |
| Joff | | 0.876555 | | 48.346423 | |
| Joff | | 0.938101 | | 49.553786 | |
| Joff | | 0.968497 | | 44.910649 | |
| Joff | | 0.99684 | | 44.399498 | |
| Joff | | 1.007469 | | 52.334765 | |
| Joff | | 1.008224 | | 49.281707 | |
| Joff | | 1.05477 | | 44.36685 | |
| Joff | | 1.06996 | | 49.513114 | |
| Joff | | 1.082954 | | 49.47072 | |
| Joff | | 1.091899 | | 52.534427 | |
| Joff | | 1.096033 | | 43.622154 | |
| Joff | | 1.096348 | | 49.406845 | |
| Joff | | 1.097794 | | 48.907039 | |
| Joff | | 1.103304 | | 49.445139 | |
| Joff | | 1.123357 | | 43.165314 | |
| Joff | | 1.129047 | | 45.029789 | |
| Joff | | 1.134988 | | 52.061635 | |
| Joff | | 1.136465 | | 49.426576 | |
| Joff | | 1.146155 | | 48.213799 | |
| Joff | | 1.156517 | | 52.056563 | |
| Joff | | 1.164116 | | 52.060856 | |
| Joff | | 1.17 | | 42.43 | |
| Joff | | 1.176462 | | 48.70137 | |
| Joff | | 1.181664 | | 47.477272 | |
| Joff | | 1.26 | | 42.53 | |
| Joff | | 1.296751 | | 45.130101 | |
| Joff | | 1.359805 | | 43.796234 | |
| Joff | | 1.367222 | | 44.859444 | |
| Joff | | 1.374879 | | 49.444353 | |
| Joff | | 1.39769 | | 45.125203 | |
| Joff | | 1.400818 | | 43.572746 | |
| Joff | | 1.412222 | | 49.165278 | |
| Joff | | 1.422276 | | 43.610059 | |
| Joff | | 1.422896 | | 43.592443 | |
| Joff | | 1.426393 | | 43.479712 | |
| Joff | | 1.433799 | | 48.369977 | |
| Joff | | 1.439909 | | 43.619374 | |
| Joff | | 1.440814 | | 43.127215 | |
| Joff | | 1.453732 | | 43.60119 | |
| Joff | | 1.45682 | | 43.583754 | |
| Joff | | 1.460261 | | 43.903801 | |
| Joff | | 1.462961 | | 43.62552 | |
| Joff | | 1.46333 | | 43.623464 | |
| Joff | | 1.464233 | | 43.605502 | |
| Joff | | 1.477303 | | 43.541799 | |
| Joff | | 1.47971 | | 43.60948 | |
| Joff | | 1.506111 | | 43.553056 | |
| Joff | | 1.506557 | | 43.593496 | |
| Joff | | 1.520563 | | 43.501373 | |
| Joff | | 1.520575 | | 43.501345 | |
| Joff | | 1.531507 | | 43.311935 | |
| Joff | | 1.54 | | 42.08 | |
| Joff | | 1.561667 | | 49.135278 | |
| Joff | | 1.602709 | | 50.717169 | |
| Joff | | 1.617343 | | 50.566433 | |
| Joff | | 1.660556 | | 43.438056 | |
| Joff | | 1.671939 | | 46.798945 | |
| Joff | | 1.68 | | 41.54 | |
| Joff | | 1.722935 | | 45.124511 | |
| Joff | | 1.727898 | | 45.053513 | |
| Joff | | 1.738789 | | 48.583706 | |
| Joff | | 1.740642 | | 49.00428 | |
| Joff | | 1.78 | | 42.17 | |
| Joff | | 1.78 | | 42.08 | |
| Joff | | 1.786397 | | 43.747008 | |
| Joff | | 1.787267 | | 49.154979 | |
| Joff | | 1.79 | | 41.54 | |
| Joff | | 1.8645 | | 50.743931 | |
| Joff | | 1.892759 | | 48.7175 | |
| Joff | | 1.915413 | | 48.987617 | |
| Joff | | 1.922403 | | 49.025394 | |
| Joff | | 1.922662 | | 49.025238 | |
| Joff | | 1.99415 | | 47.151956 | |
| Joff | | 1.994567 | | 48.780045 | |
| Joff | | 1.995819 | | 48.946332 | |
| Joff | | 1.99754 | | 48.774757 | |
| Joff | | 2.017074 | | 48.770394 | |
| Joff | | 2.017696 | | 47.909004 | |
| Joff | | 2.026858 | | 48.77203 | |
| Joff | | 2.042414 | | 48.777513 | |
| Joff | | 2.050586 | | 47.224362 | |
| Joff | | 2.053611 | | 48.854722 | |
| Joff | | 2.08196 | | 42.94047 | |
| Joff | | 2.092499 | | 49.035706 | |
| Joff | | 2.093339 | | 42.898501 | |
| Joff | | 2.09603 | | 48.896063 | |
| Joff | | 2.09925 | | 47.970505 | |
| Joff | | 2.1 | | 41.41 | |
| Joff | | 2.105973 | | 49.01543 | |
| Joff | | 2.107593 | | 48.513201 | |
| Joff | | 2.119009 | | 48.81921 | |
| Joff | | 2.134388 | | 48.908887 | |
| Joff | | 2.138354 | | 43.742345 | |
| Joff | | 2.154772 | | 48.906976 | |
| Joff | | 2.155193 | | 44.045144 | |
| Joff | | 2.16802 | | 48.992545 | |
| Joff | | 2.171918 | | 49.070937 | |
| Joff | | 2.18021 | | 44.65107 | |
| Joff | | 2.184438 | | 48.678953 | |
| Joff | | 2.187143 | | 48.869731 | |
| Joff | | 2.198755 | | 48.702868 | |
| Joff | | 2.198766 | | 48.496872 | |
| Joff | | 2.19939 | | 43.3012 | |
| Joff | | 2.221592 | | 48.819763 | |
| Joff | | 2.23112 | | 48.724554 | |
| Joff | | 2.239339 | | 48.910555 | |
| Joff | | 2.256184 | | 48.925209 | |
| Joff | | 2.263064 | | 48.846819 | |
| Joff | | 2.267711 | | 48.921444 | |
| Joff | | 2.27 | | 41.9 | |
| Joff | | 2.27 | | 42.26 | |
| Joff | | 2.271111 | | 49.148889 | |
| Joff | | 2.27572 | | 49.138699 | |
| Joff | | 2.278254 | | 43.104413 | |
| Joff | | 2.280923 | | 48.86984 | |
| Joff | | 2.282758 | | 48.912278 | |
| Joff | | 2.283835 | | 48.838084 | |
| Joff | | 2.286806 | | 48.840035 | |
| Joff | | 2.290632 | | 48.820373 | |
| Joff | | 2.294607 | | 48.520363 | |
| Joff | | 2.30996 | | 48.762473 | |
| Joff | | 2.310883 | | 48.900792 | |
| Joff | | 2.317336 | | 48.851166 | |
| Joff | | 2.321389 | | 48.789444 | |
| Joff | | 2.321593 | | 48.865856 | |
| Joff | | 2.328935 | | 48.664878 | |
| Joff | | 2.331151 | | 48.774218 | |
| Joff | | 2.331262 | | 48.976288 | |
| Joff | | 2.337196 | | 48.830717 | |
| Joff | | 2.342705 | | 48.823316 | |
| Joff | | 2.346326 | | 48.681628 | |
| Joff | | 2.351704 | | 48.893442 | |
| Joff | | 2.352765 | | 48.871811 | |
| Joff | | 2.363242 | | 48.870427 | |
| Joff | | 2.366389 | | 48.875278 | |
| Joff | | 2.366451 | | 48.874836 | |
| Joff | | 2.366994 | | 48.804456 | |
| Joff | | 2.372403 | | 48.872337 | |
| Joff | | 2.378415 | | 48.812804 | |
| Joff | | 2.382701 | | 48.706573 | |
| Joff | | 2.388374 | | 48.886418 | |
| Joff | | 2.39 | | 42.17 | |
| Joff | | 2.395017 | | 48.9696 | |
| Joff | | 2.396111 | | 43.171944 | |
| Joff | | 2.396389 | | 43.171944 | |
| Joff | | 2.397299 | | 48.855555 | |
| Joff | | 2.39844 | | 48.87233 | |
| Joff | | 2.39846 | | 48.87227 | |
| Joff | | 2.4 | | 41.45 | |
| Joff | | 2.400633 | | 48.868408 | |
| Joff | | 2.409486 | | 49.190967 | |
| Joff | | 2.410931 | | 48.868578 | |
| Joff | | 2.41121 | | 48.824364 | |
| Joff | | 2.411574 | | 48.805988 | |
| Joff | | 2.41636 | | 48.875809 | |
| Joff | | 2.417494 | | 48.895406 | |
| Joff | | 2.421686 | | 48.769886 | |
| Joff | | 2.423459 | | 48.870364 | |
| Joff | | 2.426309 | | 42.61738 | |
| Joff | | 2.428808 | | 48.857994 | |
| Joff | | 2.431862 | | 48.860473 | |
| Joff | | 2.433901 | | 48.810812 | |
| Joff | | 2.435262 | | 48.884844 | |
| Joff | | 2.439988 | | 48.811416 | |
| Joff | | 2.448788 | | 48.783366 | |
| Joff | | 2.44889 | | 42.60333 | |
| Joff | | 2.45 | | 42.23 | |
| Joff | | 2.45086 | | 42.76314 | |
| Joff | | 2.4534 | | 42.49343 | |
| Joff | | 2.456111 | | 48.860278 | |
| Joff | | 2.46581 | | 48.67298 | |
| Joff | | 2.473563 | | 49.288452 | |
| Joff | | 2.473611 | | 48.610832 | |
| Joff | | 2.476313 | | 48.820928 | |
| Joff | | 2.481101 | | 49.265972 | |
| Joff | | 2.481855 | | 48.858473 | |
| Joff | | 2.482154 | | 48.806695 | |
| Joff | | 2.496034 | | 48.820725 | |
| Joff | | 2.501872 | | 42.807803 | |
| Joff | | 2.501944 | | 48.944722 | |
| Joff | | 2.502674 | | 48.854582 | |
| Joff | | 2.506728 | | 47.758923 | |
| Joff | | 2.51 | | 42.17 | |
| Joff | | 2.519601 | | 48.06126 | |
| Joff | | 2.520039 | | 48.805282 | |
| Joff | | 2.530379 | | 42.621675 | |
| Joff | | 2.546111 | | 48.733611 | |
| Joff | | 2.559768 | | 48.66221 | |
| Joff | | 2.571424 | | 48.836607 | |
| Joff | | 2.572972 | | 48.606562 | |
| Joff | | 2.586758 | | 42.421834 | |
| Joff | | 2.590593 | | 44.364391 | |
| Joff | | 2.590662 | | 48.948574 | |
| Joff | | 2.602878 | | 43.895815 | |
| Joff | | 2.60738 | | 48.217568 | |
| Joff | | 2.63 | | 41.96 | |
| Joff | | 2.63 | | 42.17 | |
| Joff | | 2.638611 | | 44.4025 | |
| Joff | | 2.675 | | 42.474167 | |
| Joff | | 2.698302 | | 48.548258 | |
| Joff | | 2.699907 | | 48.866586 | |
| Joff | | 2.7 | | 42.1 | |
| Joff | | 2.704122 | | 49.794298 | |
| Joff | | 2.734043 | | 50.153453 | |
| Joff | | 2.743784 | | 50.290195 | |
| Joff | | 2.77252 | | 42.507431 | |
| Joff | | 2.805007 | | 42.669421 | |
| Joff | | 2.813557 | | 49.304566 | |
| Joff | | 2.82 | | 42.05 | |
| Joff | | 2.837221 | | 36.478778 | |
| Joff | | 2.843889 | | 48.713889 | |
| Joff | | 2.871944 | | 43.270833 | |
| Joff | | 2.879377 | | 50.677878 | |
| Joff | | 2.893589 | | 42.689877 | |
| Joff | | 2.89407 | | 50.461404 | |
| Joff | | 2.923073 | | 36.736919 | |
| Joff | | 2.93851 | | 43.21419 | |
| Joff | | 2.93927 | | 42.49481 | |
| Joff | | 2.961078 | | 43.563858 | |
| Joff | | 2.961438 | | 36.672267 | |
| Joff | | 2.978879 | | 50.573618 | |
| Joff | | 2.99 | | 42.45 | |
| Joff | | 3.006098 | | 36.802582 | |
| Joff | | 3.011686 | | 43.183264 | |
| Joff | | 3.012196 | | 43.184759 | |
| Joff | | 3.02087 | | 42.549675 | |
| Joff | | 3.036084 | | 50.623803 | |
| Joff | | 3.044722 | | 50.621389 | |
| Joff | | 3.052922 | | 42.538483 | |
| Joff | | 3.055414 | | 50.620057 | |
| Joff | | 3.06197 | | 43.66402 | |
| Joff | | 3.0644 | | 45.82462 | |
| Joff | | 3.06853 | | 45.7502 | |
| Joff | | 3.070158 | | 50.61517 | |
| Joff | | 3.075455 | | 50.226669 | |
| Joff | | 3.089777 | | 48.469173 | |
| Joff | | 3.099829 | | 50.657933 | |
| Joff | | 3.101819 | | 43.112207 | |
| Joff | | 3.10204 | | 50.381728 | |
| Joff | | 3.145872 | | 50.718105 | |
| Joff | | 3.151511 | | 45.679602 | |
| Joff | | 3.155526 | | 50.733357 | |
| Joff | | 3.158042 | | 46.972355 | |
| Joff | | 3.166111 | | 42.441944 | |
| Joff | | 3.221576 | | 43.337952 | |
| Joff | | 3.222215 | | 50.570215 | |
| Joff | | 3.227114 | | 50.543049 | |
| Joff | | 3.236944 | | 45.341667 | |
| Joff | | 3.245369 | | 45.842482 | |
| Joff | | 3.274823 | | 43.28491 | |
| Joff | | 3.276531 | | 43.269288 | |
| Joff | | 3.287368 | | 48.194567 | |
| Joff | | 3.295833 | | 48.559722 | |
| Joff | | 3.366711 | | 49.334807 | |
| Joff | | 3.366743 | | 49.334831 | |
| Joff | | 3.43313 | | 43.5732 | |
| Joff | | 3.434352 | | 43.605894 | |
| Joff | | 3.436389 | | 43.635278 | |
| Joff | | 3.534806 | | 43.320125 | |
| Joff | | 3.5475 | | 44.537222 | |
| Joff | | 3.601649 | | 47.789337 | |
| Joff | | 3.671298 | | 50.108453 | |
| Joff | | 3.700236 | | 49.555945 | |
| Joff | | 3.810864 | | 43.723638 | |
| Joff | | 3.837875 | | 43.537528 | |
| Joff | | 3.851322 | | 43.601812 | |
| Joff | | 3.861725 | | 43.605287 | |
| Joff | | 3.861815 | | 43.620155 | |
| Joff | | 3.867399 | | 43.612137 | |
| Joff | | 3.87672 | | 43.61077 | |
| Joff | | 3.886405 | | 43.656057 | |
| Joff | | 3.90177 | | 43.610916 | |
| Joff | | 3.923463 | | 43.64338 | |
| Joff | | 3.9384 | | 44.2 | |
| Joff | | 3.940189 | | 43.534005 | |
| Joff | | 3.95198 | | 45.01016 | |
| Joff | | 3.96281 | | 45.64042 | |
| Joff | | 3.968056 | | 44.070833 | |
| Joff | | 4.004105 | | 45.725964 | |
| Joff | | 4.023958 | | 43.724788 | |
| Joff | | 4.029821 | | 47.289673 | |
| Joff | | 4.032301 | | 44.060687 | |
| Joff | | 4.035459 | | 45.993674 | |
| Joff | | 4.037142 | | 45.968124 | |
| Joff | | 4.060833 | | 45.621667 | |
| Joff | | 4.071176 | | 44.134905 | |
| Joff | | 4.073611 | | 43.655556 | |
| Joff | | 4.085903 | | 48.333617 | |
| Joff | | 4.088523 | | 48.328067 | |
| Joff | | 4.088792 | | 48.327953 | |
| Joff | | 4.0894 | | 45.54921 | |
| Joff | | 4.09295 | | 45.72929 | |
| Joff | | 4.119139 | | 44.111007 | |
| Joff | | 4.147078 | | 43.74227 | |
| Joff | | 4.168204 | | 47.016649 | |
| Joff | | 4.168587 | | 44.581032 | |
| Joff | | 4.169167 | | 44.168333 | |
| Joff | | 4.187642 | | 44.260595 | |
| Joff | | 4.221852 | | 47.119058 | |
| Joff | | 4.251883 | | 43.79545 | |
| Joff | | 4.30543 | | 43.8362 | |
| Joff | | 4.310246 | | 43.839007 | |
| Joff | | 4.320902 | | 45.573699 | |
| Joff | | 4.338605 | | 44.716989 | |
| Joff | | 4.350504 | | 45.465245 | |
| Joff | | 4.354858 | | 48.9597 | |
| Joff | | 4.372094 | | 43.957447 | |
| Joff | | 4.375972 | | 46.674556 | |
| Joff | | 4.380693 | | 44.393484 | |
| Joff | | 4.383005 | | 43.848448 | |
| Joff | | 4.395 | | 44.016389 | |
| Joff | | 4.396766 | | 43.840764 | |
| Joff | | 4.436862 | | 43.679545 | |
| Joff | | 4.441584 | | 43.861092 | |
| Joff | | 4.558433 | | 43.975292 | |
| Joff | | 4.57343 | | 44.183193 | |
| Joff | | 4.577197 | | 44.082893 | |
| Joff | | 4.603145 | | 43.682584 | |
| Joff | | 4.676148 | | 43.667826 | |
| Joff | | 4.679059 | | 45.82033 | |
| Joff | | 4.683381 | | 44.199813 | |
| Joff | | 4.698602 | | 44.757104 | |
| Joff | | 4.70718 | | 43.95353 | |
| Joff | | 4.707721 | | 43.523608 | |
| Joff | | 4.708855 | | 44.376952 | |
| Joff | | 4.725047 | | 44.227396 | |
| Joff | | 4.75 | | 46.106389 | |
| Joff | | 4.758333 | | 44.100278 | |
| Joff | | 4.764444 | | 45.872222 | |
| Joff | | 4.779044 | | 43.813278 | |
| Joff | | 4.795737 | | 45.45792 | |
| Joff | | 4.806678 | | 45.772442 | |
| Joff | | 4.81037 | | 45.732968 | |
| Joff | | 4.8123 | | 43.644036 | |
| Joff | | 4.826858 | | 45.763489 | |
| Joff | | 4.846235 | | 43.792697 | |
| Joff | | 4.852335 | | 45.771419 | |
| Joff | | 4.853874 | | 43.879029 | |
| Joff | | 4.855485 | | 43.786738 | |
| Joff | | 4.86422 | | 45.71918 | |
| Joff | | 4.866232 | | 44.338124 | |
| Joff | | 4.86726 | | 45.8092 | |
| Joff | | 4.87135 | | 43.68899 | |
| Joff | | 4.881774 | | 45.590337 | |
| Joff | | 4.882364 | | 44.087953 | |
| Joff | | 4.958333 | | 44.939205 | |
| Joff | | 4.975756 | | 45.7475 | |
| Joff | | 4.981385 | | 45.833959 | |
| Joff | | 4.981723 | | 45.834127 | |
| Joff | | 5.036535 | | 45.913969 | |
| Joff | | 5.06485 | | 45.049713 | |
| Joff | | 5.087942 | | 43.641023 | |
| Joff | | 5.088312 | | 45.081687 | |
| Joff | | 5.131389 | | 45.866944 | |
| Joff | | 5.140517 | | 43.639912 | |
| Joff | | 5.14144 | | 44.361197 | |
| Joff | | 5.190308 | | 44.234724 | |
| Joff | | 5.236284 | | 45.594555 | |
| Joff | | 5.241163 | | 45.616878 | |
| Joff | | 5.254917 | | 43.444115 | |
| Joff | | 5.263672 | | 46.74986 | |
| Joff | | 5.311351 | | 44.277505 | |
| Joff | | 5.325508 | | 45.165206 | |
| Joff | | 5.337916 | | 43.383533 | |
| Joff | | 5.342612 | | 45.896453 | |
| Joff | | 5.386978 | | 45.188389 | |
| Joff | | 5.387239 | | 43.302492 | |
| Joff | | 5.387936 | | 43.301721 | |
| Joff | | 5.452778 | | 43.641667 | |
| Joff | | 5.497222 | | 43.297778 | |
| Joff | | 5.530323 | | 43.228736 | |
| Joff | | 5.586091 | | 46.663612 | |
| Joff | | 5.652088 | | 43.190152 | |
| Joff | | 5.66387 | | 43.6314 | |
| Joff | | 5.670449 | | 43.971336 | |
| Joff | | 5.704956 | | 36.147167 | |
| Joff | | 5.71795 | | 45.165025 | |
| Joff | | 5.740452 | | 45.173741 | |
| Joff | | 5.803889 | | 36.800278 | |
| Joff | | 5.81862 | | 44.25787 | |
| Joff | | 5.86058 | | 45.653431 | |
| Joff | | 5.900344 | | 43.467596 | |
| Joff | | 5.91603 | | 43.71497 | |
| Joff | | 5.92062 | | 43.8049 | |
| Joff | | 5.927741 | | 43.137703 | |
| Joff | | 5.93061 | | 43.46261 | |
| Joff | | 5.949457 | | 43.131908 | |
| Joff | | 5.977815 | | 45.344735 | |
| Joff | | 5.998065 | | 43.275667 | |
| Joff | | 6.04706 | | 46.15538 | |
| Joff | | 6.071647 | | 47.419391 | |
| Joff | | 6.116065 | | 43.090993 | |
| Joff | | 6.118447 | | 45.976897 | |
| Joff | | 6.124275 | | 43.1019 | |
| Joff | | 6.17893 | | 43.9778 | |
| Joff | | 6.18395 | | 44.06773 | |
| Joff | | 6.194827 | | 43.34441 | |
| Joff | | 6.197283 | | 46.182733 | |
| Joff | | 6.199932 | | 48.703814 | |
| Joff | | 6.200035 | | 48.703807 | |
| Joff | | 6.202806 | | 46.185265 | |
| Joff | | 6.23586 | | 44.0907 | |
| Joff | | 6.344777 | | 43.134407 | |
| Joff | | 6.427153 | | 43.806343 | |
| Joff | | 6.449703 | | 43.551578 | |
| Joff | | 6.48143 | | 43.529289 | |
| Joff | | 6.488173 | | 44.559657 | |
| Joff | | 6.52318 | | 43.27363 | |
| Joff | | 6.567101 | | 36.690757 | |
| Joff | | 6.581528 | | 36.340031 | |
| Joff | | 6.62665 | | 44.26182 | |
| Joff | | 6.62665 | | 44.26188 | |
| Joff | | 6.671982 | | 52.368389 | |
| Joff | | 6.69439 | | 44.23189 | |
| Joff | | 6.738166 | | 43.436297 | |
| Joff | | 6.80136 | | 43.95792 | |
| Joff | | 6.82013 | | 46.47713 | |
| Joff | | 6.89508 | | 43.50415 | |
| Joff | | 6.903096 | | 36.884429 | |
| Joff | | 6.975208 | | 47.4889 | |
| Joff | | 6.975306 | | 47.488914 | |
| Joff | | 6.978598 | | 43.719671 | |
| Joff | | 6.996238 | | 43.567605 | |
| Joff | | 7.011065 | | 43.562557 | |
| Joff | | 7.1162 | | 43.93769 | |
| Joff | | 7.12269 | | 43.726166 | |
| Joff | | 7.16148 | | 43.76455 | |
| Joff | | 7.16803 | | 43.85442 | |
| Joff | | 7.1746 | | 43.94429 | |
| Joff | | 7.18119 | | 44.03416 | |
| Joff | | 7.19444 | | 44.21392 | |
| Joff | | 7.243144 | | 43.783496 | |
| Joff | | 7.266494 | | 43.718533 | |
| Joff | | 7.30576 | | 44.02932 | |
| Joff | | 7.310603 | | 44.400652 | |
| Joff | | 7.31257 | | 44.11919 | |
| Joff | | 7.319722 | | 44.995 | |
| Joff | | 7.34162 | | 48.73984 | |
| Joff | | 7.434544 | | 48.097109 | |
| Joff | | 7.4775 | | 43.769444 | |
| Joff | | 7.479588 | | 48.460601 | |
| Joff | | 7.528001 | | 47.637972 | |
| Joff | | 7.5376 | | 47.1984 | |
| Joff | | 7.597112 | | 45.07224 | |
| Joff | | 7.615672 | | 45.083057 | |
| Joff | | 7.636916 | | 48.389206 | |
| Joff | | 7.649465 | | 45.751232 | |
| Joff | | 7.75076 | | 48.58923 | |
| Joff | | 7.956065 | | 36.284946 | |
| Joff | | 8.038067 | | 45.087942 | |
| Joff | | 8.312393 | | 46.240002 | |
| Joff | | 8.428293 | | 44.281944 | |
| Joff | | 8.523374 | | 49.85516 | |
| Joff | | 8.526936 | | 49.846066 | |
| Joff | | 8.608416 | | 45.736002 | |
| Joff | | 8.76552 | | 46.15618 | |
| Joff | | 8.79631 | | 44.432071 | |
| Joff | | 8.913378 | | 49.137066 | |
| Joff | | 8.914032 | | 49.136406 | |
| Joff | | 8.932699 | | 44.411493 | |
| Joff | | 8.94244 | | 44.40882 | |
| Joff | | 8.95092 | | 45.83895 | |
| Joff | | 8.95216 | | 45.88392 | |
| Joff | | 8.9534 | | 45.92889 | |
| Joff | | 8.998018 | | 44.401214 | |
| Joff | | 8.998404 | | 44.394808 | |
| Joff | | 9 | | 43.03333 | |
| Joff | | 9.01525 | | 45.83807 | |
| Joff | | 9.01784 | | 45.928 | |
| Joff | | 9.071492 | | 45.835504 | |
| Joff | | 9.092525 | | 45.74055 | |
| Joff | | 9.097509 | | 44.394888 | |
| Joff | | 9.191571 | | 45.473058 | |
| Joff | | 9.191698 | | 45.615948 | |
| Joff | | 9.196628 | | 44.383408 | |
| Joff | | 9.225283 | | 45.476927 | |
| Joff | | 9.24704 | | 45.518465 | |
| Joff | | 9.449169 | | 45.708241 | |
| Joff | | 9.50172 | | 42.26506 | |
| Joff | | 9.55 | | 42.26667 | |
| Joff | | 9.623056 | | 44.246667 | |
| Joff | | 9.640168 | | 45.571978 | |
| Joff | | 9.994345 | | 44.097334 | |
| Joff | | 10.073122 | | 45.815838 | |
| Joff | | 10.123795 | | 50.073849 | |
| Joff | | 10.257153 | | 45.508456 | |
| Joff | | 10.262867 | | 43.871028 | |
| Joff | | 10.279255 | | 44.606525 | |
| Joff | | 10.320726 | | 44.804394 | |
| Joff | | 10.362363 | | 42.76334 | |
| Joff | | 10.366667 | | 46.46667 | |
| Joff | | 10.601357 | | 36.403885 | |
| Joff | | 10.709242 | | 45.742844 | |
| Joff | | 10.723487 | | 45.548328 | |
| Joff | | 10.76567 | | 42.919667 | |
| Joff | | 10.828014 | | 43.872706 | |
| Joff | | 10.830005 | | 43.878075 | |
| Joff | | 10.962264 | | 45.416336 | |
| Joff | | 10.980583 | | 43.832882 | |
| Joff | | 11.027197 | | 44.532763 | |
| Joff | | 11.185618 | | 46.668627 | |
| Joff | | 11.229004 | | 44.53528 | |
| Joff | | 11.229444 | | 43.821111 | |
| Joff | | 11.267649 | | 43.885229 | |
| Joff | | 11.318889 | | 46.509444 | |
| Joff | | 11.322014 | | 44.499609 | |
| Joff | | 11.338333 | | 46.496944 | |
| Joff | | 11.348433 | | 46.440934 | |
| Joff | | 11.374774 | | 43.752877 | |
| Joff | | 11.4471 | | 43.87698 | |
| Joff | | 11.559757 | | 43.733214 | |
| Joff | | 11.599893 | | 43.607485 | |
| Joff | | 11.61691 | | 43.600287 | |
| Joff | | 11.665556 | | 46.716389 | |
| Joff | | 11.795422 | | 45.653198 | |
| Joff | | 11.818344 | | 43.703497 | |
| Joff | | 11.830417 | | 45.399338 | |
| Joff | | 11.92853 | | 45.435415 | |
| Joff | | 12.012387 | | 45.411075 | |
| Joff | | 12.012575 | | 45.411163 | |
| Joff | | 12.062322 | | 45.120284 | |
| Joff | | 12.138246 | | 45.486017 | |
| Joff | | 12.176435 | | 44.831558 | |
| Joff | | 12.185383 | | 44.693798 | |
| Joff | | 12.224938 | | 46.12788 | |
| Joff | | 12.225121 | | 46.128021 | |
| Joff | | 12.391503 | | 41.996844 | |
| Joff | | 12.423745 | | 41.930528 | |
| Joff | | 12.443333 | | 41.808056 | |
| Joff | | 12.4592 | | 41.885304 | |
| Joff | | 12.473547 | | 41.909833 | |
| Joff | | 12.490176 | | 41.825167 | |
| Joff | | 12.495371 | | 41.894681 | |
| Joff | | 12.496724 | | 41.853425 | |
| Joff | | 12.531492 | | 44.079803 | |
| Joff | | 12.535123 | | 41.933291 | |
| Joff | | 12.548783 | | 42.525909 | |
| Joff | | 12.637624 | | 41.857058 | |
| Joff | | 12.661118 | | 41.79621 | |
| Joff | | 12.663185 | | 41.999874 | |
| Joff | | 13.226108 | | 46.056263 | |
| Joff | | 13.230104 | | 42.988737 | |
| Joff | | 13.66744 | | 45.753509 | |
| Joff | | 13.672719 | | 42.842957 | |
| Joff | | 13.911944 | | 40.711389 | |
| Joff | | 14.030005 | | 42.368393 | |
| Joff | | 14.146312 | | 42.505416 | |
| Joff | | 14.196223 | | 42.483396 | |
| Joff | | 14.284722 | | 40.858056 | |
| Joff | | 14.447972 | | 45.33032 | |
| Joff | | 15.026054 | | 37.619382 | |
| Joff | | 15.040029 | | 40.963692 | |
| Joff | | 15.169154 | | 40.140644 | |
| Joff | | 15.268646 | | 37.835819 | |
| Joff | | 15.29211 | | 37.853714 | |
| Joff | | 16.208318 | | 39.303695 | |
| Joff | | 16.258239 | | 38.37332 | |
| Joff | | 16.483001 | | 39.754183 | |
| Joff | | 17.646667 | | 40.772222 | |
| Joff | | 18.174167 | | 40.362222 | |
| Joff | | 24.137197 | | 35.20245 | |
| Joff | | 24.139236 | | 35.461647 | |
| Joff | | 25.135757 | | 35.328011 | |
| Joff | | 25.38912 | | 35.314262 | |
| Joff | | 26.056944 | | 44.586667 | |
| Joff | | 26.066667 | | 38.566667 | |
| Joff | | 26.116667 | | 38.533333 | |
| Joff | | 26.946388 | | 39.541389 | |
| Joff | | 27.136944 | | 38.321111 | |
| Joff | | 27.195204 | | 38.456003 | |
| Joff | | 27.328889 | | 37.151944 | |
| Joff | | 28.21 | | 36.43 | |
| Joff | | 28.243333 | | 36.8225 | |
| Joff | | 28.2588 | | 36.8536 | |
| Joff | | 29.089722 | | 40.971667 | |
| Joff | | 30.477993 | | 36.405556 | |
| Joff | | 30.625957 | | 36.876849 | |
| Joff | | 30.978151 | | 36.929383 | |
| Joff | | 31.390181 | | 36.766778 | |
| Joff | | 32.015833 | | 36.545833 | |
| Joff | | 32.973431 | | 35.046082 | |
| Joff | | 34.7999 | | 32.11891 | |
| Joff | | 35.204845 | | 31.773373 | |
| Joff | | 36.143056 | | 36.223889 | |
| Joff | | 36.282037 | | 41.324247 | |
| Joff | | 40.839636 | | 43.08305 | |
| Joff | | 43.6092 | | 41.654702 | |
| Joff | | 69.42 | | 34.5 | |
| Joff | | 69.5 | | 35.25 | |
| Joff | | 69.83 | | 32.68 | |
| Joff | | 70.02 | | 33.93 | |
| Joff | | 70.3 | | 33 | |
| Joff | | 70.83 | | 34.75 | |
| Joff | | 70.832174 | | 31.721057 | |
| Joff | | 70.8737 | | 33.1099 | |
| Joff | | 71.35 | | 35.1 | |
| Joff | | 71.730833 | | 34.145278 | |
| Joff | | 71.83 | | 35.32 | |
| Joff | | 71.88 | | 35.2 | |
| Joff | | 72 | | 34.5 | |
| Joff | | 72.58 | | 35.33 | |
| Joff | | 72.913176 | | 34.208172 | |
| Joff | | 73.080561 | | 33.571031 | |
| Joff | | 73.42 | | 33.92 | |
| Joff | | 73.519201 | | 33.645638 | |
| Joff | | 74.395561 | | 31.56629 | |
| Joff | | 74.82 | | 34.08 | |
| Joff | | 77 | | 33 | |
| Joff | | 77.225967 | | 31.72253 | |
| Joff | | 77.491183 | | 10.237898 | |
| Joff | | 81.093611 | | 29.621111 | |
| Joff | | 82.120278 | | 29.5075 | |
| Joff | | 82.183056 | | 29.285 | |
| Joff | | 85.29 | | 28.85 | |
| Joff | | 89.663889 | | 27.447778 | |
| Joff | | 91.12 | | 29.65 | |
| Joff | | 91.73 | | 29.83 | |
| Joff | | 93.07 | | 29.04 | |
| Joff | | 94.36 | | 29.63 | |
| Joff | | 94.8653 | | 29.9894 | |
| Joff | | 95.3197 | | 29.9906 | |
| Joff | | 95.32 | | 29.99 | |
| Joff | | 95.33 | | 29.32 | |
| Joff | | 95.76 | | 29.85 | |
| Joff | | 96.91 | | 30.05 | |
| Joff | | 97.46 | | 28.66 | |
| Joff | | 97.466916 | | 28.661276 | |
| Joff | | 98.7936 | | 31.2697 | |
| Joff | | 98.819722 | | 28.186111 | |
| Joff | | 98.82 | | 31.21 | |
| Joff | | 98.84583 | | 28.35583 | |
| Joff | | 98.864722 | | 28.285278 | |
| Joff | | 98.90833 | | 29.6375 | |
| Joff | | 98.911543 | | 28.48611 | |
| Joff | | 99.28 | | 28.71 | |
| Joff | | 99.28717 | | 27.177166 | |
| Joff | | 99.416689 | | 26.453601 | |
| Joff | | 99.706013 | | 27.82308 | |
| Joff | | 99.79 | | 28.93 | |
| Joff | | 99.79929 | | 28.932073 | |
| Joff | | 99.9078 | | 29.1081 | |
| Joff | | 99.958012 | | 25.670153 | |
| Joff | | 100.17643 | | 26.56023 | |
| Joff | | 100.195084 | | 26.993265 | |
| Joff | | 100.222124 | | 25.584234 | |
| Joff | | 100.229945 | | 26.876096 | |
| Joff | | 100.298406 | | 29.037008 | |
| Joff | | 100.31 | | 30.93 | |
| Joff | | 100.311366 | | 30.93917 | |
| Joff | | 100.85195 | | 27.2822 | |
| Joff | | 101.27 | | 27.92 | |
| Joff | | 101.279419 | | 27.928607 | |
| Joff | | 101.5 | | 27.42 | |
| Joff | | 101.5 | | 29 | |
| Joff | | 101.507299 | | 29.000343 | |
| Joff | | 101.85 | | 26.68 | |
| Joff | | 101.855074 | | 26.683211 | |
| Joff | | 101.89063 | | 30.878627 | |
| Joff | | 101.96 | | 30.05 | |
| Joff | | 101.96483 | | 30.055305 | |
| Joff | | 102.234618 | | 29.914155 | |
| Joff | | 102.36 | | 30.99 | |
| Joff | | 102.364372 | | 30.99903 | |
| Joff | | 102.54 | | 30.91 | |
| Joff | | 102.593465 | | 25.869611 | |
| Joff | | 102.758313 | | 30.066708 | |
| Joff | | 102.81 | | 30.36 | |
| Joff | | 102.81464 | | 30.368117 | |
| Joff | | 104.2 | | 35.86 | |
| Jgra | | -10.5931 | | 9.7261 | |
| Jgra | | -8.74 | | 42.214 | |
| Jgra | | -4.85 | | 37.89 | |
| Jgra | | -4.71 | | 37.66 | |
| Jgra | | -4.405 | | 36.727 | |
| Jgra | | -4.39591 | | 36.73234 | |
| Jgra | | -3.982637 | | 5.351959 | |
| Jgra | | -1.42 | | 39.87 | |
| Jgra | | -0.68 | | 39.42 | |
| Jgra | | -0.6 | | 39.55 | |
| Jgra | | -0.54 | | 38.33 | |
| Jgra | | -0.46 | | 38.41 | |
| Jgra | | -0.38 | | 39.5 | |
| Jgra | | -0.13 | | 38.62 | |
| Jgra | | -0.07 | | 38.59 | |
| Jgra | | 0.07 | | 38.77 | |
| Jgra | | 2.12 | | 41.4 | |
| Jgra | | 2.71 | | 39.8 | |
| Jgra | | 2.71361 | | 39.76527 | |
| Jgra | | 7.02482 | | 43.5895 | |
| Jgra | | 7.16148 | | 43.76455 | |
| Jgra | | 7.18636 | | 43.72314 | |
| Jgra | | 26.73333 | | 37.75 | |
| Jgra | | 29.95 | | 31.204 | |
| Jgra | | 29.950001 | | 31.200001 | |
| Jgra | | 36.098055 | | 10.610833 | |
| Jgra | | 36.098167 | | 10.610833 | |
| Jgra | | 36.863333 | | 6.293333 | |
| Jgra | | 37.008333 | | 12.5 | |
| Jgra | | 37.083333 | | 18.766667 | |
| Jgra | | 37.1 | | 18.766667 | |
| Jgra | | 37.633333 | | 11.483333 | |
| Jgra | | 37.816667 | | 8.983333 | |
| Jgra | | 37.866667 | | 8.933333 | |
| Jgra | | 37.9396 | | 13.21223 | |
| Jgra | | 38.116667 | | 5.816667 | |
| Jgra | | 38.15 | | 5.616667 | |
| Jgra | | 38.5 | | 7.133333 | |
| Jgra | | 38.835 | | 14.278333 | |
| Jgra | | 38.9 | | 14.233333 | |
| Jgra | | 38.916667 | | 8.366667 | |
| Jgra | | 38.916667 | | 8.25 | |
| Jgra | | 38.983333 | | 8.75 | |
| Jgra | | 38.9885 | | 8.741833 | |
| Jgra | | 39.033333 | | 15.333333 | |
| Jgra | | 39.08 | | 8.67 | |
| Jgra | | 39.133333 | | 13.483333 | |
| Jgra | | 39.583333 | | 13.5 | |
| Jgra | | 39.616667 | | 13.3 | |
| Jgra | | 40.640167 | | 7.489167 | |
| Jgra | | 40.95 | | 9.1 | |
| Jgra | | 41.783333 | | 9.383333 | |
| Jgra | | 41.916667 | | 9.466667 | |
| Jgra | | 42.016667 | | 9.4 | |
| Jgra | | 42.083333 | | 9.333333 | |
| Jgra | | 42.083333 | | 9.416667 | |
| Jgra | | 42.1 | | 9.283333 | |
| Jgra | | 42.116667 | | 9.35 | |
| Jgra | | 42.15 | | 9.233333 | |
| Jgra | | 42.15 | | 18.966667 | |
| Jgra | | 42.4 | | 18.183333 | |
| Jgra | | 42.411111 | | 18.203333 | |
| Jgra | | 43.666667 | | 17.666667 | |
| Jgra | | 43.9 | | 15.5 | |
| Jgra | | 44.00555 | | 14.46889 | |
| Jgra | | 44.15 | | 13.233333 | |
| Jgra | | 44.15 | | 13.23333 | |
| Jgra | | 45.95 | | 13.52 | |
| Jgra | | 45.96611 | | 13.51722 | |
| Jgra | | 51.99889 | | 15.83778 | |
| Jgra | | 51.99917 | | 15.83806 | |
| Jgra | | 51.99917 | | 15.83778 | |
| Jgra | | 52.00 | | 15.83 | |
| Jgra | | 52.00 | | 15.83333 | |
| Jgra | | 52.006944 | | 15.838611 | |
| Jgra | | 52.20167 | | 15.65194 | |
| Jgra | | 52.95 | | 16.63 | |
| Jgra | | 52.95 | | 16.65 | |
| Jgra | | 52.95 | | 16.633333 | |
| Jgra | | 52.95 | | 16.63333 | |
| Jgra | | 52.9565 | | 16.6429 | |
| Jgra | | 52.96667 | | 16.65 | |
| Jgra | | 53.00 | | 16.65 | |
| Jgra | | 53.0025 | | 16.6573 | |
| Jgra | | 53.00417 | | 16.66222 | |
| Jgra | | 53.03333 | | 16.65 | |
| Jgra | | 53.03333 | | 16.63333 | |
| Jgra | | 53.05 | | 16.65 | |
| Jgra | | 53.054056 | | 16.662139 | |
| Jgra | | 53.61611 | | 16.76361 | |
| Jgra | | 53.9 | | 17.05 | |
| Jgra | | 54.066667 | | 17.166667 | |
| Jgra | | 54.31667 | | 17.1 | |
| Jgra | | 57.65 | | 23.25 | |
| Jgra | | 57.75 | | 23.166667 | |
| Jgra | | 67.00 | | 30.2 | |
| Jgra | | 69.83 | | 32.68 | |
| Jgra | | 73.498803 | | 20.753351 | |
| Jgra | | 73.498813 | | 20.75324 | |
| Jgra | | 73.498993 | | 20.754217 | |
| Jgra | | 73.499024 | | 20.752604 | |
| Jgra | | 73.735091 | | 20.576297 | |
| Jgra | | 73.93 | | 33.53 | |
| Jgra | | 74.83 | | 33.08 | |
| Jgra | | 75.17 | | 33.00 | |
| Jgra | | 76.514923 | | 9.528766 | |
| Jgra | | 79.303085 | | 12.95976 | |
| Jgra | | 81.00 | | 26.75 | |
| Jgra | | 85.29 | | 28.85 | |
| Jgra | | 87.82778 | | 23.10667 | |
| Jgra | | 89.878372 | | 27.502928 | |
| Jgra | | 91.579628 | | 27.440434 | |
| Jgra | | 91.579722 | | 27.440426 | |
| Jgra | | 94.36 | | 29.63 | |
| Jgra | | 98.28167 | | 45.4225 | |
| Jgra | | 99.79929 | | 28.932073 | |
| Jgra | | 99.9189 | | 29.0758 | |
| Jgra | | 99.92 | | 29.08 | |
| Jgra | | 100.85195 | | 27.2822 | |
| Jgra | | 102.110335 | | 26.890689 | |
| Jgra | | 102.700013 | | 25.012526 | |
| Jgra | | 103.400348 | | 24.808853 | |
| Jgra | | 113.34 | | 23.12 | |
| Jgra | | 116.21 | | 40.02 | |
| Jgra | | 118.089767 | | 24.479669 | |
| Jgra | | 119.306406 | | 26.076106 | |
| Jgra | | 120.144167 | | 23.005833 | |
| Jgra | | 121.495 | | 25.128333 | |
| Jgra | | 123.78248 | | 24.344410 | |
| Jgra | | 139.434636 | | 35.731804 | |
| Jmul | | -9.433333 | | 7.783333 | |
| Jmul | | -3.982637 | | 5.351959 | |
| Jmul | | 3.31154 | | 6.728195 | |
| Jmul | | 3.438929 | | 6.978858 | |
| Jmul | | 3.442503 | | 7.200674 | |
| Jmul | | 3.838333 | | 7.38 | |
| Jmul | | 3.894829 | | 7.44308 | |
| Jmul | | 3.9 | | 7.383333 | |
| Jmul | | 73.18 | | 22.32 | |
| Jmul | | 75.153 | | 13.625658 | |
| Jmul | | 77.944428 | | 30.47 | |
| Jmul | | 77.967675 | | 30.282881 | |
| Jmul | | 77.97496 | | 30.286517 | |
| Jmul | | 81 | | 26.75 | |
| Jmul | | 91.7194 | | 24.6583 | |
| Jmul | | 97.683333 | | 23.816667 | |
| Jmul | | 98.28167 | | 45.4225 | |
| Jmul | | 100.683333 | | 17.533333 | |
| Jmul | | 105.593167 | | 20.35 | |
| Jmul | | 105.89858 | | 14.044507 | |
| Jmul | | 108.833 | | 11.383 | |
| Jmul | | 111.013498 | | 21.586309 | |
| Jmul | | 121.3 | | 25 | |
| Jsam | | 102.421888 | | 15.56908 | |
| Jsam | | 79.904093 | | 7.066798 | |
| Jsam | | 113.915043 | | 22.22147 | |
| Jsam | | 113.984253 | | 22.411013 | |
| Jsam | | 71.50693 | | 30.265293 | |
| Jsam | | 80.427978 | | 16.325053 | |
| Jsam | | 78.531967 | | 17.490085 | |
| Jsam | | 79.062963 | | 14.71979 | |
| Jsam | | 76.841961 | | 8.648399 | |
| Jsam | | 113.481452 | | 23.431485 | |
| Jsam | | 113.979935 | | 22.23934 | |
| Jsam | | 76.16089 | | 10.549138 | |
| Jsam | | 100.301229 | | 5.355657 | |
| Jsam | | 101.60585 | | 3.147558 | |
| Jsam | | 72.971246 | | 19.194346 | |
| Jsam | | 101.780948 | | 2.991949 | |
| Jsam | | 100.416052 | | 5.403506 | |
| Jsam | | 100.473188 | | 5.175678 | |
| Jsam | | 72.971213 | | 19.194362 | |
| Jsam | | 77.879689 | | 11.434431 | |
| Jsam | | 72.971182 | | 19.194319 | |
| Jsam | | 100.306181 | | 5.357223 | |
| Jsam | | 78.239455 | | 29.771853 | |
| Jsam | | 82.663044 | | 19.895032 | |
| Jsam | | 72.971276 | | 19.194301 | |
| Jsam | | 83.31173 | | 21.818596 | |
| Jsam | | 76.375937 | | 10.776729 | |
| Jsam | | 100.306766 | | 5.354769 | |
| Jsam | | 88.128529 | | 25.630928 | |
| Jsam | | 76.2966 | | 10.0382 | |
| Jsam | | 82.140915 | | 22.079655 | |
| Jsam | | 72.971654 | | 19.194262 | |
| Jsam | | 114.023684 | | 22.451447 | |
| Jsam | | 114.023634 | | 22.451659 | |
| Jsam | | 74.36688 | | 31.528963 | |
| Jsam | | 73.683381 | | 18.578767 | |
| Jsam | | 74.641491 | | 28.993026 | |
| Jsam | | 76.971859 | | 29.410687 | |
| Jsam | | 77.743889 | | 8.728889 | |
| Jsam | | 77.689795 | | 28.957297 | |
| Jsam | | 72.904167 | | 19.113889 | |
| Jsam | | 80.358397 | | 26.469937 | |
| Jsam | | 78.165392 | | 9.132852 | |
| Jsam | | 75.634399 | | 25.41498 | |
| Jsam | | 74.736084 | | 26.300086 | |
| Jsam | | 85.177476 | | 25.539709 | |
| Jsam | | 73.013343 | | 26.240937 | |
| Jsam | | 78.041833 | | 27.445029 | |
| Jsam | | 73.007525 | | 24.695424 | |
| Jsam | | 83.139604 | | 18.631574 | |
| Jsam | | 80.552299 | | 17.202909 | |
| Jsam | | 77.329384 | | 28.345287 | |
| Jsam | | 75.351189 | | 31.328526 | |
| Jsam | | 88.338889 | | 22.755556 | |
| Jsam | | 83.468829 | | 25.853403 | |
| Jsam | | 74.510358 | | 15.880867 | |
| Jsam | | 77.68428 | | 12.99368 | |
| Jsam | | 76.158958 | | 29.032291 | |
| Jsam | | 77.011642 | | 29.245888 | |
| Jsam | | 83.249056 | | 18.937554 | |
| Jsam | | 77.689795 | | 28.957272 | |
| Jsam | | 80.251419 | | 13.099405 | |
| Jsam | | 75.811812 | | 26.880991 | |
| Jsam | | 77.010703 | | 28.491052 | |
| Jsam | | 78.479901 | | 17.531249 | |
| Jsam | | 77.740177 | | 13.044693 | |
| Jsam | | 78.434514 | | 24.681272 | |
| Jsam | | 75.32932 | | 30.477069 | |
| Jsam | | 77.133267 | | 28.694628 | |
| Jsam | | 75.768333 | | 18.991944 | |
| Jsam | | 75.51228 | | 29.470529 | |
| Jsam | | 77.335218 | | 28.571986 | |
| Jsam | | 79.003579 | | 28.565309 | |
| Jsam | | 77.325906 | | 28.419047 | |
| Jsam | | 76.600357 | | 28.896505 | |
| Jsam | | 80.15983 | | 25.82476 | |
| Jsam | | 77.074465 | | 28.627343 | |
| Jsam | | 76.131958 | | 10.8005 | |
| Jsam | | 75.130466 | | 12.282823 | |
| Jsam | | 85.51683 | | 25.190693 | |
| Jsam | | 79.913824 | | 23.126618 | |
| Jsam | | 76.365278 | | 26.054167 | |
| Jsam | | 73.974738 | | 18.577382 | |
| Jsam | | 88.38205 | | 22.884104 | |
| Jsam | | 77.071944 | | 27.573611 | |
| Jsam | | 73.168986 | | 22.294867 | |
| Jsam | | 80.357983 | | 26.408148 | |
| Jsam | | 75.768094 | | 22.551779 | |
| Jsam | | 78.140231 | | 20.419992 | |
| Jsam | | 73.753948 | | 20.003461 | |
| Jsam | | 88.609255 | | 27.329962 | |
| Jsam | | 77.078796 | | 28.705484 | |
| Jsam | | 77.108143 | | 28.422462 | |
| Jsam | | 78.199374 | | 26.210251 | |
| Jsam | | 80.937592 | | 26.938244 | |
| Jsam | | 77.078774 | | 28.705538 | |
| Jsam | | 74.712222 | | 29.664722 | |
| Jsam | | 77.402575 | | 23.267865 | |
| Jsam | | 79.041052 | | 21.103406 | |
| Jsam | | 80.907492 | | 26.807312 | |
| Jsam | | 77.285145 | | 28.862568 | |
| Jsam | | 74.554142 | | 27.937068 | |
| Jsam | | 80.034106 | | 13.129868 | |
| Jsam | | 77.059693 | | 29.879388 | |
| Jsam | | 77.702926 | | 12.977658 | |
| Jsam | | 85.300557 | | 23.390581 | |
| Jsam | | 78.029122 | | 27.220098 | |
| Jsam | | 75.746779 | | 21.917448 | |
| Jsam | | 72.540345 | | 23.121413 | |
| Jsam | | 77.396314 | | 28.49671 | |
| Jsam | | 75.565333 | | 31.342112 | |
| Jsam | | 75.281027 | | 28.885808 | |
| Jsam | | 88.429337 | | 22.601313 | |
| Jsam | | 77.105921 | | 28.92657 | |
| Jsam | | 80.495476 | | 26.548031 | |
| Jsam | | 77.28425 | | 28.68481 | |
| Jsam | | 78.044062 | | 27.581033 | |
| Jsam | | 79.856808 | | 18.040904 | |
| Jsam | | 74.605303 | | 30.964218 | |
| Jsam | | 85.23108 | | 25.59473 | |
| Jsam | | 79.004557 | | 26.778788 | |
| Jsam | | 71.658952 | | 24.908568 | |
| Jsam | | 77.019462 | | 28.599862 | |
| Jsam | | 76.617263 | | 28.187259 | |
| Jsam | | 86.474276 | | 23.812691 | |
| Jsam | | 76.31438 | | 12.309758 | |
| Jsam | | 75.908447 | | 28.802881 | |
| Jsam | | 77.338899 | | 28.691594 | |
| Jsam | | 77.115025 | | 28.707433 | |
| Jsam | | 72.836341 | | 21.155566 | |
| Jsam | | 72.769053 | | 21.2009 | |
| Jsam | | 78.113896 | | 29.92691 | |
| Jsam | | 74.953139 | | 30.210715 | |
| Jsam | | 77.08433 | | 28.633266 | |
| Jsam | | 86.99803 | | 25.249402 | |
| Jsam | | 81.863235 | | 25.455009 | |
| Jsam | | 80.938281 | | 26.881999 | |
| Jsam | | 73.020551 | | 22.930945 | |
| Jsam | | 77.423144 | | 28.623795 | |
| Jsam | | 85.350606 | | 26.081788 | |
| Jsam | | 77.095848 | | 28.47948 | |
| Jsam | | 80.231287 | | 13.091546 | |
| Jsam | | 77.379592 | | 28.576194 | |
| Jsam | | 78.338095 | | 18.874595 | |
| Jsam | | 77.634413 | | 13.019531 | |
| Jsam | | 77.038611 | | 28.516389 | |
| Jsam | | 69.889707 | | 21.603738 | |
| Jsam | | 76.706772 | | 30.810425 | |
| Jsam | | 72.520158 | | 23.075794 | |
| Jsam | | 80.262962 | | 12.995456 | |
| Jsam | | 75.901124 | | 29.364452 | |
| Jsam | | 76.440567 | | 20.989124 | |
| Jsam | | 80.19966 | | 12.985727 | |
| Jsam | | 72.902022 | | 19.091402 | |
| Jsam | | 80.933212 | | 26.948164 | |
| Jsam | | 76.953784 | | 27.797258 | |
| Jsam | | 77.050953 | | 28.427294 | |
| Jsam | | 77.703327 | | 28.968997 | |
| Jsam | | 81.668349 | | 19.589537 | |
| Jsam | | 77.634708 | | 12.928828 | |
| Jsam | | 70.779027 | | 22.307462 | |
| Jsam | | 77.6928 | | 29.46572 | |
| Jsam | | 76.190205 | | 29.508914 | |
| Jsam | | 77.568703 | | 13.149569 | |
| Jsam | | 72.529898 | | 23.044032 | |
| Jsam | | 77.683595 | | 13.014426 | |
| Jsam | | 75.843058 | | 25.131629 | |
| Jsam | | 83.253611 | | 26.456667 | |
| Jsam | | 77.938688 | | 27.194859 | |
| Jsam | | 77.408411 | | 28.676838 | |
| Jsam | | 79.366443 | | 13.973816 | |
| Jsam | | 77.657494 | | 12.821659 | |
| Jsam | | 78.213246 | | 26.208363 | |
| Jsam | | 79.324426 | | 21.395854 | |
| Jsam | | 76.569435 | | 9.559245 | |
| Jsam | | 81.60426 | | 27.580636 | |
| Jsam | | 86.604554 | | 24.812377 | |
| Jsam | | 81.957352 | | 22.737466 | |
| Jsam | | 78.769722 | | 28.838611 | |
| Jsam | | 77.739755 | | 12.791558 | |
| Jsam | | 73.452646 | | 18.676417 | |
| Jsam | | 76.364271 | | 15.162648 | |
| Jsam | | 77.600385 | | 13.012808 | |
| Jsam | | 77.674183 | | 12.923359 | |
| Jsam | | 77.666061 | | 12.738639 | |
| Jsam | | 90.366119 | | 23.838795 | |
| Jsam | | 80.575961 | | 24.699574 | |
| Jsam | | 80.195039 | | 13.035417 | |
| Jsam | | 77.650855 | | 13.010074 | |
| Jsam | | 80.177216 | | 16.08276 | |
| Jsam | | 73.891254 | | 18.474493 | |
| Jsam | | 77.425071 | | 28.701979 | |
| Jsam | | 77.435423 | | 28.651216 | |
| Jsam | | 77.103508 | | 28.68084 | |
| Jsam | | 80.171344 | | 26.573239 | |
| Jsam | | 73.224002 | | 22.340303 | |
| Jsam | | 84.370783 | | 24.753731 | |
| Jsam | | 88.297872 | | 22.682891 | |
| Jsam | | 73.344934 | | 28.003252 | |
| Jsam | | 88.042575 | | 23.956661 | |
| Jsam | | 78.08322 | | 27.947976 | |
| Jsam | | 77.133367 | | 28.694702 | |
| Jsam | | 76.153191 | | 28.601767 | |
| Jsam | | 76.413147 | | 29.805358 | |
| Jsam | | 77.360251 | | 28.397193 | |
| Jsam | | 77.583954 | | 12.885438 | |
| Jsam | | 81.706723 | | 18.075099 | |
| Jsam | | 77.602461 | | 12.930527 | |
| Jsam | | 75.084293 | | 14.373905 | |
| Jsam | | 80.119512 | | 14.362502 | |
| Jsam | | 82.53522 | | 25.609063 | |
| Jsam | | 77.131292 | | 28.467466 | |
| Jsam | | 93.600914 | | 27.087406 | |
| Jsam | | 72.783412 | | 24.47963 | |
| Jsam | | 77.121111 | | 28.711667 | |
| Jsam | | 77.835083 | | 10.332013 | |
| Jsam | | 71.4086 | | 21.233945 | |
| Jsam | | 77.391116 | | 23.21859 | |
| Jsam | | 70.052378 | | 22.485364 | |
| Jsam | | 77.02746 | | 11.054247 | |
| Jsam | | 86.958351 | | 23.684977 | |
| Jsam | | 76.642222 | | 12.351944 | |
| Jsam | | 77.620214 | | 12.886627 | |
| Jsam | | 75.861914 | | 30.913495 | |
| Jsam | | 83.354394 | | 26.763572 | |
| Jsam | | 77.576772 | | 14.693288 | |
| Jsam | | 77.298769 | | 28.7349 | |
| Jsam | | 72.565849 | | 23.081596 | |
| Jsam | | 76.992825 | | 8.511951 | |
| Jsam | | 77.317121 | | 28.671709 | |
| Jsam | | 87.23855 | | 23.570522 | |
| Jsam | | 88.602066 | | 24.131737 | |
| Jsam | | 74.592702 | | 26.470077 | |
| Jsam | | 86.754067 | | 21.385063 | |
| Jsam | | 76.306738 | | 10.37151 | |
| Jsam | | 83.296585 | | 26.040956 | |
| Jsam | | 88.253538 | | 24.104429 | |
| Jsam | | 77.418674 | | 28.686819 | |
| Jsam | | 77.490201 | | 28.685534 | |
| Jsam | | 77.010173 | | 28.468193 | |
| Jsam | | 81.006961 | | 26.850789 | |
| Jsam | | 77.492361 | | 28.703231 | |
| Jsam | | 79.957344 | | 23.162367 | |
| Jsam | | 77.645823 | | 12.95806 | |
| Jsam | | 81.007212 | | 26.971715 | |
| Jsam | | 77.324366 | | 28.491687 | |
| Jsam | | 86.181058 | | 22.777264 | |
| Jsam | | 74.478549 | | 17.311363 | |
| Jsam | | 87.306691 | | 22.35361 | |
| Jsam | | 73.795659 | | 18.443279 | |
| Jsam | | 73.108703 | | 18.549794 | |
| Jsam | | 79.490669 | | 13.013315 | |
| Jsam | | 83.435888 | | 26.754055 | |
| Jsam | | 73.05397 | | 26.293001 | |
| Jsam | | 84.504028 | | 26.808361 | |
| Jsam | | 80.147568 | | 12.888421 | |
| Jsam | | 82.773724 | | 24.198704 | |
| Jsam | | 75.408201 | | 28.489412 | |
| Jsam | | 73.88427 | | 18.524855 | |
| Jsam | | 77.314314 | | 28.373375 | |
| Jsam | | 80.368823 | | 26.446958 | |
| Jsam | | 76.596237 | | 28.883954 | |
| Jsam | | 77.041667 | | 28.633333 | |
| Jsam | | 85.309459 | | 23.38593 | |
| Jsam | | 73.922306 | | 18.464542 | |
| Jsam | | 84.858229 | | 22.232591 | |
| Jsam | | 72.866547 | | 19.031761 | |
| Jsam | | 77.070278 | | 28.586667 | |
| Jsam | | 76.452873 | | 28.351509 | |
| Jsam | | 90.306207 | | 23.887806 | |
| Jsam | | 77.18488 | | 28.707461 | |
| Jsam | | 79.379506 | | 10.342292 | |
| Jsam | | 73.053256 | | 26.335176 | |
| Jsam | | 77.300127 | | 29.44087 | |
| Jsam | | 78.029118 | | 27.220073 | |
| Jsam | | 78.5425 | | 17.471944 | |
| Jsam | | 77.960837 | | 27.20217 | |
| Jsam | | 77.04897 | | 28.475275 | |
| Jsam | | 77.270643 | | 28.696238 | |
| Jsam | | 72.645889 | | 23.206857 | |
| Jsam | | 73.724905 | | 18.500377 | |
| Jsam | | 77.641494 | | 13.022118 | |
| Jsam | | 82.955029 | | 25.347343 | |
| Jsam | | 77.490561 | | 12.952587 | |
| Jsam | | 76.031868 | | 28.904226 | |
| Jsam | | 83.333468 | | 18.139076 | |
| Jsam | | 74.401218 | | 30.393847 | |
| Jsam | | 76.898078 | | 15.160517 | |
| Jsam | | 77.059416 | | 29.87952 | |
| Jsam | | 77.379554 | | 28.57624 | |
| Jsam | | 88.429504 | | 22.601578 | |
| Jsam | | 76.08687 | | 26.645807 | |
| Jsam | | 76.659009 | | 28.471627 | |
| Jsam | | 75.78384 | | 30.920979 | |
| Jsam | | 77.295465 | | 28.70305 | |
| Jsam | | 76.929195 | | 28.679533 | |
| Jsam | | 79.828514 | | 26.21339 | |
| Jsam | | 77.087922 | | 28.603661 | |
| Jsam | | 81.790218 | | 21.594834 | |
| Jsam | | 77.34457 | | 10.003014 | |
| Jsam | | 77.300127 | | 29.440869 | |
| Jsam | | 75.836617 | | 30.873459 | |
| Jsam | | 88.291964 | | 22.579761 | |
| Jsam | | 77.168752 | | 28.731377 | |
| Jsam | | 77.068062 | | 28.627186 | |
| Jsam | | 75.799075 | | 26.941427 | |
| Jsam | | 86.497917 | | 23.855458 | |
| Jsam | | 77.18944 | | 28.768393 | |
| Jsam | | 74.342859 | | 16.218383 | |
| Jsam | | 77.68729 | | 12.910295 | |
| Jsam | | 77.291152 | | 28.499588 | |
| Jsam | | 73.16394 | | 19.20706 | |
| Jsam | | 78.607063 | | 17.715731 | |
| Jsam | | 72.684555 | | 19.89625 | |
| Jsam | | 77.299235 | | 29.441907 | |
| Jsam | | 75.852124 | | 30.255195 | |
| Jsam | | 75.793209 | | 26.852622 | |
| Jsam | | 76.730911 | | 19.490004 | |
| Jsam | | 77.338329 | | 28.691534 | |
| Jsam | | 77.338684 | | 28.691659 | |
| Jsam | | 75.699996 | | 29.124687 | |
| Jsam | | 80.33739 | | 26.474039 | |
| Jsam | | 79.299958 | | 18.215948 | |
| Jsam | | 77.658735 | | 28.716853 | |
| Jsam | | 78.04326 | | 27.443706 | |
| Jsam | | 78.407961 | | 17.468536 | |
| Jsam | | 88.351845 | | 26.710163 | |
| Jsam | | 74.177391 | | 23.845463 | |
| Jsam | | 77.896927 | | 21.888172 | |
| Jsam | | 83.004694 | | 25.370851 | |
| Jsam | | 88.142212 | | 25.00528 | |
| Jsam | | 82.948447 | | 21.778578 | |
| Jsam | | 78.132513 | | 8.813488 | |
| Jsam | | 81.856367 | | 25.447371 | |
| Jsam | | 75.60286 | | 31.351713 | |
| Jsam | | 75.833149 | | 22.687689 | |
| Jsam | | 73.950494 | | 18.487037 | |
| Jsam | | 75.815702 | | 13.335045 | |
| Jsam | | 79.274187 | | 12.686459 | |
| Jsam | | 79.436417 | | 25.984903 | |
| Jsam | | 88.473537 | | 22.57676 | |
| Jsam | | 71.644001 | | 24.780678 | |
| Jsam | | 84.914923 | | 26.06607 | |
| Jsam | | 75.281037 | | 28.885797 | |
| Jsam | | 77.080522 | | 28.602138 | |
| Jsam | | 77.100108 | | 28.751686 | |
| Jsam | | 73.193753 | | 19.199975 | |
| Jsam | | 77.382778 | | 28.519444 | |
| Jsam | | 83.499094 | | 25.919314 | |
| Jsam | | 75.799461 | | 23.154638 | |
| Jsam | | 77.300716 | | 28.510906 | |
| Jsam | | 77.864243 | | 28.246569 | |
| Jsam | | 85.30055 | | 23.39057 | |
| Jsam | | 76.712861 | | 29.133506 | |
| Jsam | | 77.191313 | | 28.4927 | |
| Jsam | | 70.050833 | | 22.483889 | |
| Jsam | | 77.651652 | | 13.014164 | |
| Jsam | | 77.34938 | | 28.626278 | |
| Jsam | | 72.570674 | | 23.10272 | |
| Jsam | | 73.49858 | | 23.492934 | |
| Jsam | | 80.037121 | | 12.796966 | |
| Jsam | | 82.467308 | | 25.583206 | |
| Jsam | | 76.199565 | | 20.510227 | |
| Jsam | | 77.314762 | | 21.159205 | |
| Jsam | | 77.995922 | | 26.499272 | |
| Jsam | | 72.601656 | | 22.988664 | |
| Jsam | | 85.147232 | | 25.595314 | |
| Jsam | | 77.102314 | | 28.46444 | |
| Jsam | | 83.799682 | | 26.066532 | |
| Jsam | | 77.334254 | | 28.669333 | |
| Jsam | | 85.543889 | | 24.885 | |
| Jsam | | 80.639366 | | 16.231151 | |
| Jsam | | 77.832072 | | 12.707592 | |
| Jsam | | 73.798369 | | 18.499339 | |
| Jsam | | 76.567078 | | 9.662809 | |
| Jsam | | 71.350105 | | 21.778173 | |
| Jsam | | 77.327599 | | 28.34985 | |
| Jsam | | 87.516255 | | 25.81009 | |
| Jsam | | 72.841763 | | 25.316709 | |
| Jsam | | 76.513294 | | 28.295295 | |
| Jsam | | 77.609878 | | 13.054001 | |
| Jsam | | 72.901992 | | 19.091396 | |
| Jsam | | 78.053146 | | 27.893547 | |
| Jsam | | 73.873331 | | 25.105174 | |
| Jsam | | 75.651482 | | 20.316328 | |
| Jsam | | 73.818301 | | 18.619044 | |
| Jsam | | 77.091356 | | 22.33672 | |
| Jsam | | 84.967934 | | 25.739454 | |
| Jsam | | 74.328346 | | 29.567849 | |
| Jsam | | 75.769203 | | 31.226339 | |
| Jsam | | 76.244823 | | 28.14222 | |
| Jsam | | 88.601234 | | 23.22038 | |
| Jsam | | 73.278176 | | 21.090735 | |
| Jsam | | 83.182123 | | 25.170013 | |
| Jsam | | 77.709435 | | 29.020119 | |
| Jsam | | 77.461252 | | 23.251339 | |
| Jsam | | 75.063073 | | 29.537112 | |
| Jsam | | 77.202251 | | 10.968824 | |
| Jsam | | 77.927755 | | 9.460894 | |
| Jsam | | 88.380901 | | 22.499902 | |
| Jsam | | 75.83863 | | 30.251621 | |
| Jsam | | 77.610568 | | 12.902329 | |
| Jsam | | 83.347729 | | 25.764008 | |
| Jsam | | 77.465515 | | 9.99712 | |
| Jsam | | 84.703705 | | 19.602835 | |
| Jsam | | 88.512859 | | 22.568342 | |
| Jsam | | 77.531724 | | 28.515064 | |
| Jsam | | 76.944321 | | 17.877184 | |
| Jsam | | 80.308207 | | 21.520068 | |
| Jsam | | 85.402629 | | 26.130321 | |
| Jsam | | 82.506042 | | 25.245491 | |
| Jsam | | 75.13293 | | 12.283862 | |
| Jsam | | 72.658545 | | 23.051386 | |
| Jsam | | 79.535436 | | 12.683002 | |
| Jsam | | 74.050759 | | 16.475143 | |
| Jsam | | 77.665397 | | 13.003613 | |
| Jsam | | 77.560723 | | 12.985514 | |
| Jsam | | 77.44658 | | 28.600268 | |
| Jsam | | 78.187814 | | 16.055501 | |
| Jsam | | 77.065261 | | 28.617544 | |
| Jsam | | 76.370059 | | 26.974299 | |
| Jsam | | 82.50605 | | 25.245466 | |
| Jsam | | 81.675446 | | 21.213198 | |
| Jsam | | 77.045954 | | 28.475923 | |
| Jsam | | 72.470849 | | 23.02157 | |
| Jsam | | 75.815805 | | 30.921665 | |
| Jsam | | 78.083774 | | 27.948062 | |
| Jsam | | 72.616299 | | 22.988985 | |
| Jsam | | 81.157694 | | 20.091555 | |
| Jsam | | 75.874254 | | 30.700054 | |
| Jsam | | 77.539427 | | 12.98867 | |
| Jsam | | 77.323348 | | 28.684335 | |
| Jsam | | 88.375556 | | 22.524722 | |
| Jsam | | 76.331744 | | 26.96144 | |
| Jsam | | 81.02875 | | 26.856675 | |
| Jsam | | 79.138449 | | 12.910409 | |
| Jsam | | 73.797886 | | 18.500496 | |
| Jsam | | 78.05182 | | 13.401036 | |
| Jsam | | 83.324791 | | 26.015702 | |
| Jsam | | 86.84584 | | 23.740671 | |
| Jsam | | 82.418446 | | 17.156238 | |
| Jsam | | 79.078526 | | 21.91262 | |
| Jsam | | 76.016219 | | 12.88812 | |
| Jsam | | 77.656688 | | 13.01005 | |
| Jsam | | 75.654811 | | 15.448986 | |
| Jsam | | 83.332346 | | 27.17013 | |
| Jsam | | 78.372344 | | 17.395915 | |
| Jsam | | 80.205824 | | 12.936858 | |
| Jsam | | 86.748052 | | 21.920207 | |
| Jsam | | 77.987248 | | 10.360685 | |
| Jsam | | 76.395178 | | 10.2785 | |
| Jsam | | 88.389968 | | 22.624431 | |
| Jsam | | 77.656735 | | 13.010117 | |
| Jsam | | 86.726917 | | 21.928683 | |
| Jsam | | 87.296784 | | 23.55805 | |
| Jsam | | 75.923657 | | 30.897401 | |
| Jsam | | 77.54111 | | 12.916678 | |
| Jsam | | 76.882318 | | 12.737043 | |
| Jsam | | 80.222783 | | 13.085414 | |
| Jsam | | 77.655573 | | 13.027308 | |
| Jsam | | 75.272132 | | 23.26504 | |
| Jsam | | 77.037671 | | 28.469361 | |
| Jsam | | 76.353126 | | 27.686253 | |
| Jsam | | 79.439446 | | 16.475989 | |
| Jsam | | 76.309879 | | 9.72875 | |
| Jsam | | 76.358944 | | 10.029135 | |
| Jsam | | 79.243353 | | 11.323038 | |
| Jsam | | 76.926094 | | 9.017612 | |
| Jsam | | 79.803611 | | 28.302633 | |
| Jsam | | 73.787308 | | 18.492603 | |
| Jsam | | 73.884743 | | 18.524746 | |
| Jsam | | 77.850622 | | 13.273503 | |
| Jsam | | 81.269233 | | 26.210747 | |
| Jsam | | 82.915159 | | 18.750663 | |
| Jsam | | 78.562367 | | 17.409397 | |
| Jsam | | 83.082944 | | 27.261545 | |
| Jsam | | 85.834987 | | 20.350236 | |
| Jsam | | 77.503714 | | 13.092978 | |
| Jsam | | 74.388986 | | 15.981633 | |
| Jsam | | 86.263634 | | 23.828444 | |
| Jsam | | 88.336192 | | 22.720728 | |
| Jsam | | 78.496389 | | 17.352222 | |
| Jsam | | 76.820017 | | 30.719159 | |
| Jsam | | 80.934537 | | 26.768137 | |
| Jsam | | 82.966118 | | 26.011292 | |
| Jsam | | 72.830233 | | 19.195712 | |
| Jsam | | 82.942827 | | 26.181781 | |
| Jsam | | 84.677823 | | 26.097653 | |
| Jsam | | 73.733604 | | 18.590936 | |
| Jsam | | 88.086533 | | 24.497568 | |
| Jsam | | 77.345936 | | 28.591261 | |
| Jsam | | 87.570492 | | 21.852493 | |
| Jsam | | 87.845257 | | 24.652676 | |
| Jsam | | 88.114647 | | 22.490261 | |
| Jsam | | 85.538808 | | 25.45665 | |
| Jsam | | 88.385119 | | 22.604967 | |
| Jsam | | 77.102787 | | 28.487309 | |
| Jsam | | 81.350882 | | 18.884211 | |
| Jsam | | 77.674199 | | 12.920867 | |
| Jsam | | 76.575012 | | 18.427823 | |
| Jsam | | 77.859418 | | 12.746198 | |
| Jsam | | 82.264275 | | 19.205166 | |
| Jsam | | 73.737168 | | 18.663953 | |
| Jsam | | 77.851111 | | 9.170556 | |
| Jsam | | 84.090299 | | 18.782235 | |
| Jsam | | 79.416408 | | 18.500294 | |
| Jsam | | 80.146624 | | 12.909329 | |
| Jsam | | 103.8566 | | 13.347703 | |
| Jsam | | 80.032845 | | 16.233959 | |
| Jsam | | 88.384722 | | 22.530833 | |
| Jsam | | 77.754458 | | 29.013806 | |
| Jsam | | 88.295117 | | 22.481911 | |
| Jsam | | 77.365471 | | 28.661274 | |
| Jsam | | 85.002133 | | 24.7952 | |
| Jsam | | 86.119802 | | 20.962166 | |
| Jsam | | 72.610556 | | 23.195 | |
| Jsam | | 83.902062 | | 20.848618 | |
| Jsam | | 72.824582 | | 19.217066 | |
| Jsam | | 77.773468 | | 8.725117 | |
| Jsam | | 91.816843 | | 22.330996 | |
| Jsam | | 76.986933 | | 11.080251 | |
| Jsam | | 78.126503 | | 17.597242 | |
| Jsam | | 77.006687 | | 28.660401 | |
| Jsam | | 90.249415 | | 23.84319 | |
| Jsam | | 73.852505 | | 18.500564 | |
| Jsam | | 80.014959 | | 13.951063 | |
| Jsam | | 86.801163 | | 22.61923 | |
| Jsam | | 79.802533 | | 28.63462 | |
| Jsam | | 69.832603 | | 22.382162 | |
| Jsam | | 77.773435 | | 8.725004 | |
| Jsam | | 74.583702 | | 18.155622 | |
| Jsam | | 73.852749 | | 18.499434 | |
| Jsam | | 79.758575 | | 26.58613 | |
| Jsam | | 80.367195 | | 15.830186 | |
| Jsam | | 68.4 | | 27.3 | |
| Jsam | | 79.303085 | | 12.95976 | |
| Jsam | | 72.830131 | | 19.212029 | |
| Jsam | | 54.612322 | | 24.352862 | |
| Jsam | | 98.583333 | | 3.316667 | |
| Jsam | | 105.718333 | | 20.234167 | |
| Jsam | | 71.730833 | | 34.145278 | |
| Jsam | | 77.55 | | 11.07 | |
| Jsam | | 70.8737 | | 33.1099 | |
| Jsam | | 109.83 | | 27.2 | |
| Jsam | | 110.35 | | 25.17 | |
| Jsam | | 113.34 | | 23.12 | |
| Jsam | | 87.96518 | | 22.466857 | |
| Jsam | | 73.647734 | | 17.930729 | |
| Jsam | | 101.661763 | | 2.831642 | |
| Jsam | | 91.584969 | | 27.440608 | |
| Jsam | | 72.961845 | | 19.145052 | |
| Jsam | | 114.221938 | | 22.46837 | |
| Jsam | | 114.267514 | | 22.308119 | |
| Jsam | | 87.305078 | | 23.499449 | |
| Jsam | | 106.711196 | | 10.818933 | |
| Jsam | | 58.106273 | | 23.330965 | |
| Jsam | | 78.9989 | | 28.8898 | |
| Jsam | | 104.088668 | | 30.710589 | |
| Jsam | | 110.032046 | | 20.801294 | |
| Jsam | | 113.938254 | | 22.696199 | |
| Jsam | | 111.295924 | | 23.490446 | |
| Jsam | | 109.511923 | | 18.252625 | |
| Jsam | | 108.842336 | | 24.905949 | |
| Jsam | | 108.174498 | | 34.31924 | |
| Jsam | | 109.507052 | | 18.249731 | |
| Jsam | | 87.304842 | | 23.499627 | |
| Jsam | | 113.341527 | | 23.127041 | |
| Jsam | | 110.050532 | | 25.255089 | |
| Jsam | | 109.321003 | | 21.533749 | |
| Jsam | | 100.79926 | | 22.010889 | |
| Jsam | | 106.633387 | | 24.392902 | |
| Jsam | | 103.086573 | | 22.792186 | |
| Jsam | | 80.702788 | | 28.504358 | |
| Cay | 6.671792 | | 43.466958 | |  |
| Cay | 6.401845 | | 43.280105 | |  |
| Cay | 6.68612 | | 43.47115 | |  |
| Cay | 14.158087 | | 41.217335 | |  |
| Cay | 7.155 | | 43.67468 | |  |
| Cay | 9.147266 | | 39.200238 | |  |
| Cay | 23.513327 | | 37.929079 | |  |
| Cay | 1.7674 | | 41.24837 | |  |
| Cay | 10.58806 | | 35.73194 | |  |
| Cay | 10.56556 | | 35.87722 | |  |
| Cay | 10.59694 | | 35.89333 | |  |
| Cay | 23.455271 | | 37.498613 | |  |
| Cay | 15.2775 | | 37.859 | |  |
| Cay | 15.794861 | | 39.848676 | |  |
| Cay | 16.8715 | | 41.12807 | |  |
| Cay | 13.335998 | | 38.139395 | |  |
| Cay | 10.69779 | | 42.94545 | |  |
| Cay | 7.90502 | | 43.84027 | |  |
| Cay | 3.876716 | | 43.610769 | |  |
| Cay | 9.101827 | | 39.226949 | |  |
| Cay | 3.876716 | | 43.610769 | |  |
| Cay | 4.899333 | | 43.967247 | |  |
| Cay | 10.322571 | | 36.842468 | |  |
| Cay | 9.222264 | | 45.480078 | |  |
| Cay | 76.93173 | | 8.503426 | |  |
| Cay | 76.931718 | | 8.503432 | |  |
| Cay | 72.129513 | | 21.756184 | |  |
| Cay | 101.69438 | | 16.842333 | |  |
| Cay | 104.83518 | | 21.99679 | |  |
| Cay | 103.815184 | | 1.312574 | |  |
| Cay | 102.597866 | | 17.948669 | |  |
| Cay | 100.3321 | | 18.23387 | |  |
| Cay | 104.847178 | | 21.987235 | |  |
| Cay | 105.80994 | | 20.9678435 | |  |
