# Supplementary figures and images for: Corythauma ayyari (Insecta, Heteroptera, Tingidae) depends on its host plant to spread in Europe
Source: PLoS One. 2024 Mar 26;19(3):e0295102. doi: 10.1371/journal.pone.0295102 (PMC10965059; doi:10.1371/journal.pone.0295102)

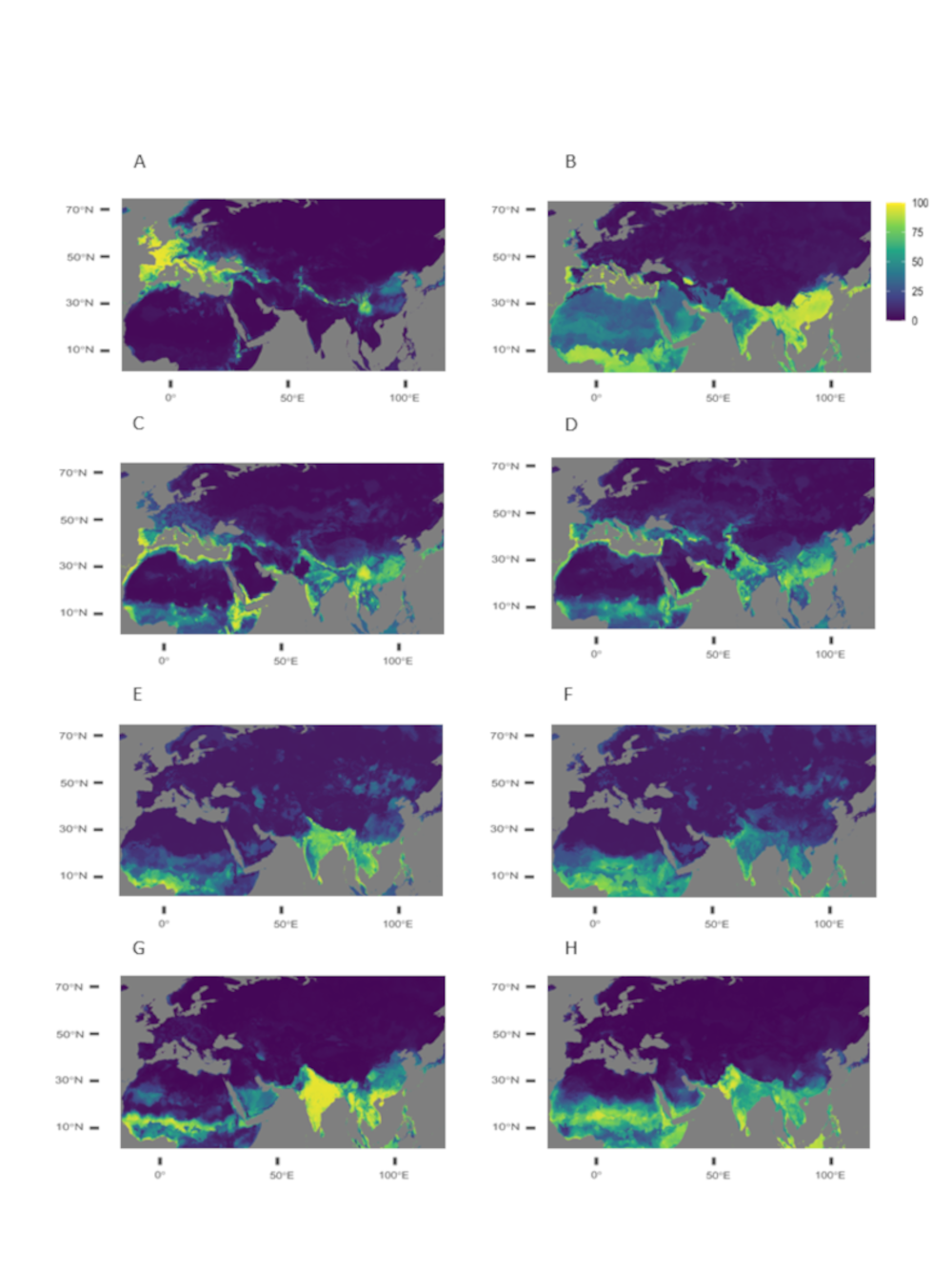

Supplement: S1 Fig — Projections of the current (on the left) and future (on the right) environmental conditions resulting from the niche modelling for Jasminum species. Ensemble projection combines all model with an arbitrary threshold up to 0.7 and uses the TSS binary metric, for the four main Jasminum species hosting C. ayyari: A, B) J. officinale; C, D) J. grandiflorum; E, F) J. multiflorum; G, H) J. sambac. (TIF) [file pone.0295102.s002.tif]
