# Supplementary material for: Corythauma ayyari (Insecta, Heteroptera, Tingidae) depends on its host plant to spread in Europe
Source: PLoS One. 2024 Mar 26;19(3):e0295102. doi: 10.1371/journal.pone.0295102 (PMC10965059; doi:10.1371/journal.pone.0295102)
Supplement: S1 Table — Correlation between species distribution using Pearson coefficient, under current condition (above) and future conditions (below). Species distributions are rasters resulting from species distribution modelling using PCA components as variables. (DOCX) [file pone.0295102.s003.docx]

| Current conditions | *J. officinale* | *J. grandiflorum* | *J. multiflorum* | *C. ayyari* |
| --- | --- | --- | --- | --- |
| *J. sambac* | -0.0982 | 0.4833 | 0.5743 | 0.4610 |
| *J. officinale* |  | 0.4107 | 0.1464 | 0.3192 |
| *J. grandiflorum* |  |  | 0.5380 | 0.4234 |
| *J. multiflorum* |  |  |  | 0.3433 |

| Future conditions | *J. officinale* | *J. grandiflorum* | *J. multiflorum* | *Cayyari* |
| --- | --- | --- | --- | --- |
| *J. sambac* | -0.0855 | 0.4323 | 0.5223 | 0.5636 |
| *J. officinale* |  | 0.4753 | 0.1577 | 0.1204 |
| *J. grandiflorum* |  |  | 0.5173 | 0.2519 |
| *J. multiflorum* |  |  |  | 0.2990 |
