# Supplementary material for: Corythauma ayyari (Insecta, Heteroptera, Tingidae) depends on its host plant to spread in Europe
Source: PLoS One. 2024 Mar 26;19(3):e0295102. doi: 10.1371/journal.pone.0295102 (PMC10965059; doi:10.1371/journal.pone.0295102)
Supplement: S2 Table — A first modelling uses only the first nine climatic variables issued from a PCA; a second modelling includes also the four Jasminum species distribution as variable. (DOCX) [file pone.0295102.s004.docx]

S2 Table.

| Variables | Contribution | Percentage | Variables | Contribution | Percentage |
| --- | --- | --- | --- | --- | --- |
| climPCA1 | 0.1415 | 14.6868 | climPCA1 | 0.0347 | 7.5161 |
| climPCA2 | 0.1195 | 12.3967 | climPCA2 | 0.0049 | 1.0618 |
| climPCA3 | 0.1074 | 11.1491 | climPCA3 | 0.0150 | 3.2587 |
| climPCA4 | 0.0499 | 5.1832 | climPCA4 | 0.0088 | 1.9154 |
| climPCA5 | 0.3717 | 38.5719 | climPCA5 | 0.1270 | 27.5346 |
| climPCA6 | 0.0634 | 6.5761 | climPCA6 | 0.0057 | 1.2249 |
| climPCA7 | 0.0760 | 7.8897 | climPCA7 | 0.0198 | 4.2983 |
| climPCA8 | 0.0177 | 1.8329 | climPCA8 | 0.0027 | 0.5824 |
| climPCA9 | 0.0165 | 1.7135 | climPCA9 | 0.0178 | 3.8575 |
|  |  |  | *j. grandiflorum* | 0.0590 | 12.8007 |
|  |  |  | *j. multiflorum* | 0.0251 | 5.4506 |
|  |  |  | *j. officinale* | 0.1051 | 22.7766 |
|  |  |  | *j. sambac* | 0.0356 | 7.7223 |
