# Supplementary material for: Corythauma ayyari (Insecta, Heteroptera, Tingidae) depends on its host plant to spread in Europe
Source: PLoS One. 2024 Mar 26;19(3):e0295102. doi: 10.1371/journal.pone.0295102 (PMC10965059; doi:10.1371/journal.pone.0295102)
Supplement: Attachment — Submitted filename: Response to reviewers.docx [file pone.0295102.s005.docx]

**Response to reviewers**

**Dear Editor,**

Here the answers of all the comments of the reviewers. We made all changes according to reviewer requests. In addition, we mention that the figures are original graphics done with the packages and the softwares used for the analyses. No need of copyrights as far as we know.

Thank you for considering our revised manuscript.

Eric

Reviewer #1: Line 1-2: I don't think that this title can be chosen based on the results presented. Invasion is also not a good term to use here (it might not happen). Spread or proliferate might be better. The fact that this species depends on its host plant seems to be known so the title does not really present the results of the study.

*Agree. I then changed the tittle with “spread” rather than “invasion”.*

Line 15: Invasiveness would need to be defined as a term or concept with references, ”potential to become widespread” might be better.

*We changed “invasiveness” by “potential to become widespread” as suggested.*

Line 20: What kind of modeling was done? Ensemble forecasting?

*We did ensemble forecasting. However, as a method it is not specified in the abstract. We specified it at the mat & met section.*

Line 20-22: Rephrase the sentence and specifically mention your species. Otherwise it reads as if it has not been considered before with other insect plant association. Or is this something really new?

*Use of host plants in niche modelling of phytophagous insects is rare, but exists. Therefore, we modified the sentence.*

Line 26: I don't think this is relevant in your study. Please give some general citations here, maybe specifically for disruptions caused by insects and/or plants.

*Agree. We tried to focus more on insects and added some more information in the paragraph.*

Line 41: Maybe it would be nice to introduce the concept of pest species (in the native range) and invasive species (non-native range) once.

*For sure! We added the concepts at the beginning of the paragraph (more appropriately).*

Line 46: Please add: introduced via trade “to Europe”. These Jasmine species are just considered a non-native species which has value as an ornamental plant and therefore should not be damaged by C. ayyari (which would be the “bad” invader)?

*Jasmine species are non-native species to Europe. As far as I know they are not considered as invasive but rather introduced. Whatever, they are damaged by C. ayyari either in the invaded and native areas. I’m not sure we understood well this last comment…*

Line 49: Are they a real problem in the native range, thus considered a pest species? If so, why are there so little occurrence records of C. ayyari? Due to no one sampling it?

*C. ayyari is considered as a pest species in India, the native range of the main host plants. Apparently, it is not seriously considered and very few specimens were recorded in Asia, the native area of both Jasmine species and C. ayyari. As apparently not clear enough, we added some more details here.*

Line 53: Which other crop species could this be?

*Added two genera on which C. ayyari has been collected.*

Line 61: The interesting bit is that biotic variables are rarely taken into account because the relationship to a species’ occurrence is often not entirely known. But in the case of plant and parasitic insect, the host plant’s (or many host plants’) occurrence(s) and (in)directly its climatic requirements should be the main factors that determine whether the insect survives, unless there are some other environmental factors that are different for the insects survival and the plants survival, e.g. predator of the insect. I am not sure looking into all future distribution model for the 4 Jasmine species alone is necessary for this study in such detail (see suggestion to cut part in the Results below).

*Well, we tried to provide most of the information for the reader to judge our results. Future projection for the Jasmine species is necessary to project C. ayyari distribution into the future. However, they are not the main topic of the study and we can cut off parts as suggested.*

Line 62: If they are strictly dependent on the host plant, why would you model only with bioclimatic variables at all? I would expect strong correlations between host plant and some climatic variables if you put them together into one model.

*Modelling with bioclimatic variables only is the most commonly done. We did so to verifying whether including biotic variable render the modelling more accuate. We could do so after getting sure biotic variables were not or weakly correlated to bioclimatic variables, as shown in the manucsript.*

Line 67: Please be more specific what these studies showed.

*Sure. A sentence was added to specify what showed each of the two studies referenced.*

Line 68-74: As said above, why model it without host plant at all if it is strictly dependent on it?

*We aim to compare the accuracy of our models with and without the biotic variables, as most of niche modelling are based on bioclimatic variables only.*

Line 78: I think it is debatable whether you want to call it invasion if it is considered a pest species in its native range already although there are so little number of occurrence points from its native range, which of course might be due to a sampling bias (too little sampling effort). Would the sampling (and any occurrence records) of C. ayyari not be correlated with Jasmine species?

*We agree on the term ‘invasion’ and therefore change it to ‘spread area’ relative to the distribution area of the host plants. We verified also before analyzing that C. ayyari known distribution was not correlated with Jasmine species distribution.*

Line 86: See comment before. Do you know whether sightings of C. ayyari were made on plants?

*No mention of particular sightings of C. ayyari in the literature. There are other specimens of C. ayyari collected, but most were collected a long time ago and thus, without accurate location. We choose to not use these specimens to avoid bias in niche modelling. This is also why this study was done, in order to evaluate species distribution based on known occurrences. We added a sentence specifying the inaccurate locations for many specimens.*

Line 116: Are Jasminum species already considered in equilibrium because they were introduced long time ago and are not spreading any longer in Europe?

*Yes, Jasmine species are known in Europe since a long time, and as such can be considered as in equilibrium. We specified it.*

Line 155-157: If C. ayyari cannot survive without its plant host, this analysis is not really useful.

*Fore sure… We then deleted this part.*

Line 164-165: That seems very little occurrences to base models on overall. Can you comment on the minimum number of occurrence records necessary for the different models you used? If this is accounted for by using the TSS and different thresholds are calculated in each model irrespectively of what the number of occurrence records is, please describe it in more detail.

*We are aware of the sample size effect and worked on it. Several authors ensure the use of small sample size does not affect model accuracy (Stockwell & Perterson 2002; Hernandez et al. 2006; Jiménez-Valverde 2020). We added into the mat & met section some explanations/justifications about the sample size effect on modeling.*

Fig.1: There is no co-occurrence in the native range? I suppose sightings were made on other host plants than Jasminum species. Do you know on what plants sightings (occurrence records) of C. ayyari were made?
Do these species not actually overlap in the native range in Asia?

*We did not check co-occurrence in native range in detail. Co-occurrence exist in non-native range, of course. Other plants were recorded as hosting C. ayyari, however, most are not georeferenced and casual (meaning probably ‘visited’ plants). Volkameria inermis and Trachelospermum jasminoides, the main two other plants mentioned do not overlap with Jasmine species in Asia.*

Line 172: Please explain the Boyce index as a means to validate model results in more detail.

*We added more detail on Boyce index in the mat & met section.*

Line 170-225: This is just describing the Figures in the supplementary material and the correlation tables (which should also go into the supplementary material) focusing on the bioclimatic variables and current and future distribution for the plants and C. ayyari separately. The main focus should rather be on comparing the current and future models excluding or including these Jasmine species as biotic variables when projecting current and future distribution C. ayyari. Try to shorten it or place text into supplementary.

*Right. We then deleted the part on Jasmine species modeling and put the correlation table into supplementary information, driving the reader to supp. Info. For Jasmine modeling.*

Line 232-236: This is the main result, incl. Fig. 2.

Line 243-244: What does “all models together” mean? The models used for the ensemble forecasting? What are these 5th, 1st and 2nd PCA components? Can you show the PCA plots?

*Yes, ‘all models together’ means ‘the ensemble forecasting’. We changed the terms as suggested. PCA components are the variables used to model the distribution of species. There were used to avoid co-linearity between variables. The disadvantage is that distribution modelling cannot be related with the original variables such as the bioclimatic ones.*

*PCA plots will be a figure grouping the nine rasters of PCA components retained… We are not sure it would contribute that much to the understanding of the analysis. Now, that can be done if necessary. Or as supplementary information? Or only the three more contributing variables?*

Line 249: If this is the case, why would you expect a high invasion potential of C. ayyari due to availability of Jasmine species in Europe? Are you assuming that in the native range (Asia) they don’t overlap but in Europe they could? It seems that very little observations of C. ayyari living on Jasmine spp have been made in Asia (judging by the low amount of occurrence records on GBIF) although the insect is considered a pest species. Due to the little number of occurrences, I would also question the niche parameter calculations for C. ayyari (line 270-272). Maybe you can discuss the quality of data in the discussion.

*We are not expecting a high invasion of C. Ayyari, but highlight the fact that it can shift on a different ecological niche in native area. According to the data and the analyses, we assume they don’t overlap in Asia but do in Europe. Of course, there may be a bias in the sample and we added some comment on the quality of the data in the discussion.* *However, Broenniman et al. (2012) used kernel density function to determine a smoothed density of occurrences. That makes moving from geographical space, where the species occur, to the multivariate environmental space, where analyses are performed, independent of sampling effort. This was added in the discussion.*

L. 267: Could you explain the Schoener’s index meaning in more detail? When do you talk of a high overlap and a low overlap? Same for similarity and equivalency.

*Sure… We added some more explanations for Schoener’s D, equivalency and similarity in the method section.*

Line 275: What does Csa mean?

*Csa, as well as Aw and Cwa (L 284-285), refer to the World map of the Köppen-Geiger climate classification (ref. 30-32), without providing any further information. We therefore deleted these three contractions but let the references to the World map cited.*

Line 278-281: It would be good to describe the life cycle of C. ayyari in more detail to see at what stage the host plant plays a role.

*For sure. We added what is known about C. ayyari biology in the discussion.*

L 284-285: What does Aw and Cwa mean?

*See line 275 comment above.*

L. 321: Can you comment on model validation a bit more? And the data availability and how this might have influenced the projections, especially C. ayyari for which data records are very low.

*We already added some information on data availability as requested for Line 164-165. However, we added some more consideration about model validation as asked.*

L. 331-333: How reliable is it to talk of a niche switch here? How dependent is it on the host plant and how dependent on the climatic conditions?

*As far as there is no niche overlap, it means the native and non-native environments are different. In this case what else than a shift from one niche to another would be? Niche modelling shows that Jasmine species is an important variable for C. ayyari distribution. Difficult to discuss the importance of climatic conditions as we used PCA components instead to avoid auto-correlations between variables. Some words on that point are added in the discussion*

L. 335-337: Do you want to discuss niche shift (comparing native and invaded area) of the plants as well?

*No. We just want to highlight that such shift is not rare as shown for different groups.*

L 342-344: If they are not host plants and only visited plants, could they still damage them? Have there been any experimental studies been done to test the “vulnerability” of these plants?

*Not any studies on plant vulnerability for C. ayyari is known. Difficult here to go further in the discussion.*

L 362-363: But C. ayyari is considered native in some of these regions if I understood correctly and considered a pest species. Why do you talk of its invasion in these regions then?

*Right. The sentence is not correct and we therefore modified to clarify this point.*

Reviewer #2: In the reviewed manuscript by Manon Durand and Eric Guilbert presented results of modeling the possibility of successful development of the invasive bug species of Tingidae, Corythauma ayyari in Europe depending on the distribution of its host plants, some species of jasmine.
The analysis of the possibilities of settling of this invasive species using special programs makes it possible to foresee the distribution of C. ayyari in the future. In general, the results appear to be quite reliable. However, the authors’ conclusions about the probability of significant suitability (up to 50%) for the habitat of the tropical species C. ayyari (lines in the text 205-214) in the territory of Central Europe seem unconvincing to me.

*Current and future suitability expressed as a percentage fit definitely above 50% in many parts of Central Europe (West above Black Sea) and also East, above Black Sea. However, not all the area is 50% suitable. It is probably not obvious considering the figures, but they can be improved for publication. Whatever, we added details into the result section considering the 50% suitable areas, and we added some consideration about the unconvincing of Central Europe as suitable area into the discussion.*

In addition, the authors' suggestion that the oligophage C. ayyari "...can damage jasmine species as well as other ornamental and/or agricultural plants, causing significant environmental and economic damage" (lines 52-44) appears to be unfounded, and this assumption follows put away

*Yet, it causes damage on host plant definitely, no publication attested its economic and environmental impact. Nothing published neither about potential damages on other plants. We then deleted this part.*

Overall, the manuscript is recommended for publication with minor corrections.
