# Supplementary material for: Corythauma ayyari (Insecta, Heteroptera, Tingidae) depends on its host plant to spread in Europe
Source: PLoS One. 2024 Mar 26;19(3):e0295102. doi: 10.1371/journal.pone.0295102 (PMC10965059; doi:10.1371/journal.pone.0295102)
Supplement: Attachment — Submitted filename: Response to reviewers.docx [file pone.0295102.s006.docx]

**Response to reviewers**

**Dear Editor,**

Here the answers of all the comments of the reviewers. We made all changes according to reviewer requests. We also added a figure (Fig 3) showing the PCA plots of the 19 climatic variables under current and future conditions, as requested.

Thank you for considering our revised manuscript.

Eric

Maybe show in a PCA-plot all variables (as arrows) and their contribution, i.e. how they place along the two PCA-axes PC1 (x-axis, highest variability explained) and PC2 (y-axis, second highest variability explained).

*Sure! It has been done in Fig 3, in the results.*

New Table S1 in the Supplementary Material is missing.

*Oops… We added it. Sorry.*

To be corrected (Lines refer to text with Track Changes)

*All corrections hereafter have been considered in a manuscript with only these track changes*

Line 133: since they WERE introduced
Line 140: as stated BY Jiménez-Valverde
Line 176: A niche equivalency and a niche similarity test (not tests)
Lines 311-313: It stated that C. ayyari develops at a temperature ranging from 22 and 31°C, AND a relative humidity ranging from 86.75 to 91.88%. Adults emerge at night and mate 24 hours after emergence. Females live between 9 to 12 days and mate WITH an average of 34 eggs.
Line 357: Jasminum in absent IN THE East of Germany (should it not say EUROPE?)
Line 368-369: Small geographic and environmental ranges CAN ALSO POSTITIVELY affect model performance
Line 423: these non-native regions
